# Characterization of the prohormone complement in cattle using genomic libraries and cleavage prediction approaches

**DOI:** 10.1186/1471-2164-10-228

**Published:** 2009-05-16

**Authors:** Bruce R Southey, Sandra L Rodriguez-Zas, Jonathan V Sweedler

**Affiliations:** 1 Department of Chemistry, University of Illinois, Urbana, IL, USA; 2 Department of Animal Sciences, University of Illinois, Urbana IL, USA

## Abstract

**Background:**

Neuropeptides are cell to cell signalling molecules that regulate many critical biological processes including development, growth and reproduction. These peptides result from the complex processing of prohormone proteins, making their characterization both challenging and resource demanding. In fact, only 42 neuropeptide genes have been empirically confirmed in cattle. Neuropeptide research using high-throughput technologies such as microarray and mass spectrometry require accurate annotation of prohormone genes and products. However, the annotation and associated prediction efforts, when based solely on sequence homology to species with known neuropeptides, can be problematic.

**Results:**

Complementary bioinformatic resources were integrated in the first survey of the cattle neuropeptide complement. Functional neuropeptide characterization was based on gene expression profiles from microarray experiments. Once a gene is identified, knowledge of the enzymatic processing allows determination of the final products. Prohormone cleavage sites were predicted using several complementary cleavage prediction models and validated against known cleavage sites in cattle and other species. Our bioinformatics approach identified 92 cattle prohormone genes, with 84 of these supported by expressed sequence tags. Notable findings included an absence of evidence for a cattle relaxin 1 gene and evidence for a cattle galanin-like peptide pseudogene. The prohormone processing predictions are likely accurate as the mammalian proprotein convertase enzymes, except for proprotein convertase subtilisin/kexin type 9, were also identified. Microarray analysis revealed the differential expression of 21 prohormone genes in the liver associated with nutritional status and 8 prohormone genes in the placentome of embryos generated using different reproductive techniques. The neuropeptide cleavage prediction models had an exceptional performance, correctly predicting cleavage in more than 86% of the prohormone sequence positions.

**Conclusion:**

A substantial increase in the number of cattle prohormone genes identified and insights into the expression profiles of neuropeptide genes were obtained from the integration of bioinformatics tools and database resources and gene expression information. Approximately 20 prohormones with no empirical evidence were detected and the prohormone cleavage sites were predicted with high accuracy. Most prohormones were supported by expressed sequence tag data and many were differentially expressed across nutritional and reproductive conditions. The complete set of cattle prohormone sequences identified and the cleavage prediction approaches are available at .

## Background

Neuropeptides are a diverse class of signalling peptides that include neurotransmitters and peptide hormones that have various paracrine, endocrine, and autocrine effects [1,2]. Neuropeptides support cell to cell communication and regulate diverse biological processes such as blood flow, synaptogenesis, memory, learning, reproduction, lactation, development, growth, feeding, behavior and cell morphology [1,2]. Only 42 neuropeptide-containing genes, appreciably fewer than the expected number, have been empirically confirmed in cattle tissues. Annotating the cattle neuropeptide complement is important as these molecules play a critical role in cattle production, health and well-being. For example, cattle neuropeptide Y (*NPY*) stimulates food intake, oxytocin stimulates smooth muscle contraction, vasopressin stimulates water re-absorption in the kidney, and ghrelin stimulates appetite and feeding activity through interactions with *NPY *and other peptides [3]. Genetic variation in cattle neuropeptide genes has been associated with variation in traits of economical importance including birth weight, average daily gain, body weight, feed conversion ratio, rib-eye area, marbling score and subcutaneous fat depth [4-7].

The annotation of neuropeptides will aid functional studies that use high-throughput transcriptomic (e.g., microarray) and proteomic (e.g., 2D gels, mass spectrometry) techniques. Several microarray platforms derived from the cattle genome and expressed sequence tag (EST) databases (e.g., NCBI Gene Expression Omnibus or GEO [8]) are available such as GEO platforms GPL2853, GPL2864, GPL3301, GPL3810, GPL6497, GPL2112, and GPL1854. These platforms include a variable number of probes that map to neuropeptide-containing genes. However, the incomplete annotation of the cattle neuropeptide complement has hindered the ability to characterize the expression profiles of neuropeptide-containing genes. Experimental confirmation of neuropeptides and experimental peptidome studies are resource intensive [2,9-11]. Although neuropeptides can be biochemically characterized using mass spectrometry, such efforts are considerably enhanced by the addition of neuropeptide-containing gene annotations that allows the association of mass spectral peaks with specific peptides [2].

Annotating the neuropeptide complement is complicated because neuropeptides are derived from larger proteins by a complex series of post-translational modifications. Translation of the neuropeptide-containing gene generates a large protein known as a preproneuroptide or preprohormone, which conceptually consists of a signal peptide region and a region that contains one or more peptides. The signal peptide is removed by signal peptidases to form the proneuropeptide or prohormone (hereinafter referred to as prohormone) [1,2,12]. The prohormone may undergo further cleavage by other proteases, notably proprotein or prohormone proteases, at basic amino acids (Arg or Lys) [1,2,12]. After cleavage, the terminal basic amino acids are typically removed by carboxylases or Arg/Lys aminopeptidases and various additional post-translational modifications (e.g., amidation, glycosylation) can occur before the final bioactive neuropeptides are produced [1,2,12]. The resulting bioactive neuropeptides are often small, typically between 3 to 40 residues long [1,2].

Prohormone processing is also highly dependent on the environment (e.g., pH), prohormone structural properties, alternative splicing, presence of specific proteases or proteases with different affinity for cleavage [1,2,12,13]. Furthermore, the presence of neuropeptides varies across species, tissues, developmental stages, and with other conditions [1,2,12]. Accordingly, experimental detection of neuropeptides in mammals has been limited to only a few species (notably human, mouse and rat) and neuropeptide families such as insulin.

There are two distinct phases in the process of annotating the neuropeptide complement of a species. The first phase requires the identification of the prohomone genes. Prohormones and neuropeptides frequently have different lengths across species and a very short conserved region that interacts with its cognate receptor [1,2]. As a consequence, the prohormone may contain large and highly variable sections that show limited homology to well-studied species. Therefore, while the prohormone gene in one species may be identified by sequence homology to another better studied species, homology alone is insufficient to accurately annotate the actual neuropeptides within the prohormone sequence. The second phase of the annotation process involves the identification of neuropeptides within the prohormone and this requires the prediction of the prohormone cleavage sites. Accurate prediction of prohormone cleavage sites and resulting peptides has been achieved using machine learning techniques such as logistic regression, artificial neural networks, support vector machines and memory-based reasoning [14-18].

The availability of the cattle genome sequence provides an unprecedented opportunity to conduct a comprehensive identification of cattle neuropeptide genes using complementary bioinformatic tools, databases and information resources. The goal of this study was to compile and characterize the cattle prohormone genes and neuropeptide complement. First, prohormone genes were located on the recently published cattle genome sequence [19] using a strategy that integrates information from complementary genomic databases [18]. This strategy addressed the situation where the initial automated annotation failed to detect genes or detected only partial sequences. Second, biological evidence for predicted prohormone genes was obtained from public genomic and EST databases. Third, the prohormone genomic census was used to accurately annotate cattle microarray platforms and subsequently to assess the presence and differential expression of neuropeptide genes in microarray experiments. Lastly, machine and statistical learning tools were applied to our database of prohormone gene sequences to predict prohormone cleavage sites and resulting peptides. The resulting catalogue also benefits neuropeptide annotation in other ungulate and mammalian livestock species that do not have sequenced genomes. Our integrative bioinformatics strategy can be applied to annotate the neuropeptide complements in other species that have comparable genomic and transcriptomic information.

## Methods

### Neuropeptide prohormone identification and characterization

To detect prohormone genes in cattle, a list of candidate prohormone genes was generated from multiple sources including public databases and the literature. First, the mammalian (primarily human, mouse, rat, and cattle) prohormone genes used by Amare et al. [15] and Tegge et al. [17] were combined. Additional prohormone genes were identified from the UniProt database release 14.0 [20] using the protein family field and a search using neuropeptide-like characteristics such as hormone or neuropeptide molecular functions. The SwePep database [21] was also used to supplement the list of candidate prohormone genes because it focuses on small peptides detected by mass spectrometry.

Candidate genes were searched for in the cattle genome Btau_3.1 assembly using the sequence alignment tool BLAST [22] following the approach described by Southey et al. [18]. The search was conducted using the NCBI BLAST standalone version 2.18 with default parameters (E-value of 10 and BLOSUM62 scoring matrix) and disabled filtering. The BLAST results from each prohormone were screened based on the alignment score and E-value to identify the most likely matches and location of the corresponding cattle prohormone gene in the genome. In addition, results were examined for multiple homologous prohormone genes that could indicate gene duplication events in the cattle genome. The protein prohormone sequences were identified within the detected genome regions using the gene parsing tool Wise2 [23]. Wise2 predicts the gene structure using a gene prediction model that includes introns and frameshift errors based on a target protein sequence and a genomic DNA sequence. The target protein sequences were selected from the candidate list with a preference for cattle or sheep genes. The genomic region encompassing the BLAST match was extended approximately 500 base pairs to the 5' and 3' ends of the match. Each predicted prohormone gene was compared to the UniProt and Entrez Gene [24] databases to assess the accuracy of the prediction based on previously reported prohormone genes. The predicted protein sequence was then compared to the corresponding published sequences using the multiple sequence alignment tool, Clustalw [25]. This step also served to confirm the suitability of the Wise2 prediction. If a suitable prediction was not obtained from the extended genomic region, protein sequences from other species were also used. Raw genomic data (including unassigned genomic regions, whole genome shotgun sequencing and trace archives) were also searched when there was no suitable BLAST match to a candidate or when the alignment to the genome assembly indicated a missing genomic region. This strategy allowed the annotation of genomic regions that were partly or not included in the assembly. The more recent Btau_4.0 assembly [26] and University of Maryland assembly 1.5 (UMD_1.5; )[27], which became available during the annotation process, were used to identify remaining prohormone genes not found in the Btau_3.1 assembly.

### Expressed sequence tag information

The comprehensive identification of prohormone genes in the cattle genome constitutes the first step toward a comprehensive characterization of the neuropeptide gene set. However, prediction of prohormone gene sequences is insufficient evidence of the actual presence and expression of these genes. Reported cattle ESTs provide independent support for prohormone genes, especially for the unpublished cattle prohormone genes. The candidate genes were searched for on the UniGene database (build #92) and information on the ESTs (number of ESTs, sequence, overlap) and tissue of expression were extracted from the database for each cattle prohormone gene. This search was complemented with searches for candidate genes on the NCBI EST (dbEST release 080107) and NCBI Nucleic databases to encompass any cattle ESTs and nucleic acid sequences that were not included in the available UniGene release.

### Transcriptomic analysis

A comprehensive characterization of neuropeptide gene expression profiles was attained by querying and analyzing two complementary resources. First, a survey of gene expression records across tissues and developmental stages available in the UniGene and EST databases was performed. This survey offered an introductory glimpse at the expression patterns of prohormone genes. However, the nature of the UniGene data-spanning experiments, most with no connecting samples, prevents the profiling and relative quantification of prohormone gene expression. To address this, a second resource, the NCBI GEO database, was inspected for informative microarray studies. Consideration was given to experiments that included at least five biological replicates per condition and two technical replicates per sample and that used a platform with at least 50% of the identified cattle prohormone genes. These requirements ensured a minimum accuracy on the detection of prohormone gene expression and precision on the profile estimates.

Two large microarray investigations met these criteria and were selected for examination of the presence of neuropeptides and for evidence of differential expression between conditions. The first, reported by Loor et al. [28], consisted of liver samples from healthy cows and those exposed to a nutritional plane conducive to ketosis. The second, reported by Everts et al. [29], consisted of placentome samples of pregnancies from calves obtained using three reproductive techniques: *in vitro *fertilization (IVF), somatic cell nuclear transfer (NT) and artificial insemination (AI). Both experiments used the same cattle microarray platform, GEO platform GPL2853, which has 13,257 70-oligomer elements printed in duplicate. The microarray platform contained 45 known cattle prohormone genes with the complete gene sequence available, two prohormone genes with only a partial sequence previously reported, and nine previously unreported prohormone genes. The platform also contained the sequence of a probe (OLIGO_09208) that spans a splice site of the torsin family 2 member A (*TOR2A*) gene. Due to the location of this splice site, this oligomer represents the *TOR2A *isoform 1 ([Swiss-Prot:A4FUH1]) that is not a prohormone and not the *TOR2A *isoform 4 (Swiss-Prot:P0C7W1), which is the prohormone that produces salusin neuropeptides in other mammals [30]. However, this microarray element was considered a probe for the prohormone gene due to possible cross-hybridization of the *TOR2A *isoform 4 to the region of the probe prior to the splice site.

The microarray data filtering, normalization and analyses used in this study were the same as described in Loor et al. [28] and Everts et al. [29], respectively. Briefly, fluorescence data processing encompassed the filtering of spots marked as unreliable by the scanning software or weak (when compared to control elements) and loess normalization before fitting a two-stage, mixed-model analysis. In the first stage, gene expression values were adjusted for global dye and microarray effects and in the second stage, the expression of each microarray element was described with a model including the effects of dye-, sample-, microarray- and experimental-specific factors. Only the patterns of prohormone gene expression across health status [28] and embryo type [29] are reported here. The statistical significance of the differential expression was adjusted for multiple testing across neuropeptide genes using the false discovery rate approach [31].

### Prediction of cleavage sites

The cleavage sites of all prohormone genes were predicted using logistic regression and artificial neural network models developed using 42 cattle prohormone sequences [17] in NeuroPred [32]. Prior to prediction, the signal peptide and known cleavage sites were identified based on experimental evidence from the UniProt record when available. When no experimental evidence was available, the signal peptide length was predicted using SignalP [33] and cleavage sites were assigned based on homology to known cleavage sites from other species.

## Results and Discussion

### Prohormone identification

There were 92 candidates for cattle prohormone genes identified from the literature and protein databases and these included 42 cattle prohormone genes with empirical evidence. The bioinformatics search identified 92 cattle prohomone genes that included a novel calcitonin gene but failed to identify one candidate. Table 1 presents the distribution of the cattle prohormone genes with complete and partial sequences that were identified in the cattle genome across the nucleic and protein resources used to detect the genes. A detailed description of the 92 prohormone genes with supporting evidence from the Entez Gene, Unigene and UniProt databases is provided [see Additional file 1]. The protein sequences of the discovered prohormone genes with cleavage sites identification is provided in the format used by NeuroPred [see Additional file 2].

**Table 1:** Distribution of the candidate prohormone genes identified in the cattle genome among public databases

		UniProt^1^			
			
Genome^2^	Nucleic^3^	Complete	Fragment	TrEMBL	Unreported^4^
Complete	UniGene	56	3	16	6
	DNA	1	0	0	2
	Unreported	1	2	0	3

Fragment	UniGene	1	0	0	0
	Pseudogene	1	0	0	0

Not found	Unreported	0	0	0	1

^1 ^Derived from information provided in the corresponding UniProt release; Complete indicates the complete prohormone is present in the SwissProt database, Fragment indicates a partial sequence in the SwissProt database, and TrEMBl indicates a complete sequence without experimental data is reported in the TrEMBL database.

^2 ^Complete, Fragment and Not found denote if the prohormone gene was completely recovered, partially recovered or there were no matches from the cattle genome, respectively.

^3 ^Denotes the resource supporting the presence of the prohormone sequence in the cattle genome: UniGene, DNA (nucleotide and EST sequences not present in UniGene), pseudogenes and unreported.

^4 ^Not previously reported (predicted or empirically confirmed) in cattle.

The initial BLAST query to the Btau_3.1 assembly indicated that 88 prohormone gene candidates were likely to be present (E-value < 10^-6^). The complete sequences of 80 cattle prohormone genes were subsequently obtained by using Wise2 with the Btau_3.1 assembly. The remaining eight candidates with strong BLAST evidence were located in the genome but had incomplete sequences in the Btau_3.1 assembly. Complete sequences for six candidates, including five that have been previously reported with complete sequences, were recovered using the recent Btau_4.0 and UMD_1.5 assembly. Of the remaining two candidates with strong BLAST evidence, the secretin gene (*SECR*) including the reported cattle secretin peptide ([Swiss-Prot:P63296]) was not recovered due to incomplete coverage of the genomic region based on the sequence available (Dr. Steven Salzberg, Dr. Liliana Florea and Finn Hanrahan, personal communication), and sequence characteristics (discussed below) suggested that galanin-like peptide gene (*GALP*) is a pseudogene (discussed below). In addition, three candidate genes, cocaine and amphetamine responsive transcript (*CART*), peptide YY (*PYY*) and seminalplasmin or peptide YY2 (*PYY2*), have published cattle sequences and were recovered using the UMB_1.5 assembly because there were no significant matches (E-value > 1) to the Btau_3.1 and Btau_4.0 assemblies.

Of the detected genes available in UniProt, 56 prohormone genes have complete and annotated sequences, 16 prohormone genes have complete sequences without annotation, four prohormone genes have complete sequences but have only been reported as fragments (three in SwissProt and one in TrEMBL), and nine new prohormone genes have complete sequences previously unreported (not reported in UniGene) in cattle (Table 1). A comparison of genomic and reported sequences showed that 14 prohormone genes had different sequences due to single nucleotide polymorphisms, two prohormone genes had undetermined amino acids, and adenylate cyclase activating polypeptide 1 (*ADCYAP1*) includes an apparently incorrect sequence. The predicted amino acid sequence of the *ADCYAP1 *prohormone gene was more consistent (higher percentage of identity and similarity) with other species than the cattle SwissProt sequence ([Swiss-Prot:Q29W19]). The available UniProt protein sequence for cattle *TOR2A *does not include the isoform corresponding to the alpha-salusin and beta-salusin neuropeptides that was found in this study.

The use of complementary nucleotide databases was critical to validate the complete set of predicted prohormone genes. There were 81 prohormone sequences with cattle ESTs in the UniGene database and two prohormone genes, relaxin 3 (*RLN3*, [GenBank:BI682322] and neuropeptide W, (*NPW*; [GenBank:DY084317]), with cattle ESTs not included in UniGene. In addition, the full DNA sequence of cattle islet amyloid polypeptide gene (*IAPP*; [GenBank:AJ675853]) was not present in UniGene. Although a cattle gonadotropin-releasing hormone 2 (*GON2*) was detected, the lack of EST data may support the finding that this gene is functionally inactive [34]. Our approach predicted adrenomedullin 2 (*ADM2*), neuromedin U (*NMU*), tuberoinfundibular 39 residue protein (*TIP39*) and tachykinin 4 (*TAC4*) prohormone genes with complete sequences but without cattle EST data. This constitutes important findings because molecular techniques that rely on EST information (e.g., the design of microarray platforms or primers when there is no genome information available) will not be able to detect these genes.

Multiple genomic matches to the candidate query sequences can uncover gene duplication events. Species-specific neuropeptide prohormone variants resulting from duplication have been reported in other mammalian species. Examples include insulin-like 4 gene (*INSL4*) in humans and chimpanzees [35], hepcidin antimicrobial peptide 2 gene (*HEPC2*) in mouse [36], and two variants of insulin gene (*INS*) found in various rodents including rat and mouse [37]. In all previous cases, the searches resulted in a single match to a single cattle prohormone, indicating that there was no support for duplicated genes or cattle-specific prohormone genes. With the exception of the calcitonin family, the examination of additional BLAST matches provided no evidence for duplicated prohormone genes in the cattle genome that were not previously expected based on homology to protein families (e.g., the insulin family). Our approach uncovered a potential duplication in the calcitonin family because it had four matches; interestingly, there are two members of the calcitonin family in human, mouse and rat. Further findings about the calcitonin family are discussed in the forthcoming calcitonin family section.

#### Galanin-like peptide (GALP) pseudogene

The evidence for a cattle *GALP *pseudogene is due to a lack of matches to cattle EST data and predicted gene structure. The matching region of cattle genome BLAST match on cattle chromosome 18 included the sequence GWTLNSAGYLLGP, which is completely conserved across mammalian *GALP *and the related galanin (*GALA*) genes. The cattle *GALA *[SwissProt: P11242] has been previously reported on cattle chromosome 29 and the match also included a larger region that is only conserved across the *GALP *sequences. Additional *GALP *matches to unassigned contigs in the recent cattle genome assemblies were detected using as query the human *GALP *genomic sequence (including exons and introns), but there was insufficient coverage to recover a complete sequence. This discovery is likely to correspond to a pseudogene because no initiation codon was found (Dr. Steven Salzberg, Dr. Liliana Florea and Finn Hanrahan, personal communication). A fragment of a sheep *GALP *prohormone sequence that also matched the same genomic location (E-value < 0.15) has been previously reported ([Swiss-Prot:A2TEF1]. However, the reliability of the sheep sequence information is questionable because the sheep *GALP *nucleic and protein sequences were identical to the rat *GALP *nucleic ([GenBank:AF188491] and protein ([Swiss-Prot:Q9QXQ6]) sequences, respectively.

#### Relaxin family

The relaxin family is a subfamily of the insulin family [35] although the family member notation varies across species. Human, mouse and rat have relaxin 1 (*RLN1*), relaxin 3 (*RLN3*), insulin-like 3 (*INSL3*), insulin-like 5 (*INSL5*) and insulin-like 6 (*INSL6*), of which rat *INSL5 *has been reported as a pseudogene [35]. Cattle *INSL3 *and *INSL6 *prohormone genes have been previously reported and the complete sequences were also recovered from the genome. The complete sequences for the genes *INSL5 *(supported by [Unigene:Bt.101509]) and *RLN3 *(supported by EST [GenBank:BI682322]) were recovered in this study, although these genes have not previously been reported in cattle.

A remarkable result is that there were no matches on the cattle genome or on the cattle ESTs to any available mammalian *RLN1 *sequence that was not attributable to another member of the relaxin family. No matches to cattle genome sequences or ESTs were identified using the sheep relaxin-like pseudo-gene ([GenBank:S60580]; [38]) and the *RLN1 *protein and mRNA sequences from camel ([GenBank:AF254739]), dog ([SwissProt:Q9TRM8]), horse ([SwissProt:P22969] and pig ([SwissProt: P01348]). A search for *RLN1 *was conducted using the trace archives from other species belonging to Cetartiodactyla clade. There was a strong match (E-value < 10^-100^) to a relaxin-like gene in the *Vicugna vicugna *(vicuña) that was very similar to the camel sequence, as expected, since both species are members of the Tylopoda subclade. In the Cetacea subclade there was a strong match (E-value < 10^-100^) to the *Tursiops truncatus *(bottlenosed dolphin). Except for a match (E-value < 10^-100^) in the sheep trace archives for the known pseudo-gene, there were no matches in any of the Ruminantia species including various deer species (such as red and fallow deer) and *Antilocapra americana *(pronghorn).

To further elucidate the possible location of a cattle relaxin 1 gene or genes, the human chromosome that contains *RLN1 *and the corresponding cattle chromosome were compared. Human *RLN1 *is located on chromosome 9 between the *INSL6 *and chromosome 9 open reading frame 46 (*C9orf46*) and the distance between these loci is approximately 170K bp. Both *INSL6 *and *C9orf46 *are located on cattle chromosome 8 but are only approximately 35 K bp apart. These results strongly suggest that the relaxin 1 gene has been lost from the cattle genome.

#### Calcitonin family

The composition of the calcitonin family is complex due to gene duplication, alternative splicing, different nomenclature, pseudogenes and partial protein sequences. The human, mouse and rat calcitonin family includes calcitonin or the calcitonin gene-related peptide 1 (*CGRP-I *or alpha-type *CGRP *or *CALCA*) and calcitonin gene-related peptide 2 (*CGRP-II *or beta-type *CGRP *or *CALCB*) genes. Alternative splicing of *CALCA*, a human calcitonin pseudogene (*CALCP*) [39] and three calcitonin-related proteins in pig [40] have been reported.

A query of the calcitonin prohormone family resulted in four matches on cattle chromosome 15 that spanned a region of 500 Kbp. These matches were supported by ESTs in four UniGene clusters; UniGene cluster Bt.29881 (including four ESTs), UniGene cluster Bt.73268 (including 1 EST), UniGene cluster Bt.14302 (including 11 ESTs), UniGene cluster Bt.60861 (including 6 ESTs) and UniGene cluster Bt.28622 (including 5 ESTs). Two of the matches were also supported by three UniProt TrEMBL calcitonin-related records with complete gene sequences in cattle: *CALCB *([Swiss-Prot:Q17Q98]), calcitonin receptor-stimulating peptide-1 ([Swiss-Prot:Q75V95]), and calcitonin-related polypeptide 3 or *CALC3 *([Swiss-Prot:Q0VBW3]). There is strong evidence suggesting that [Swiss-Prot:Q17Q98] and [Swiss-Prot:Q75V95] are the result of alternative splicing. The first 75 amino acids of these two sequences are identical, there is substantial overlap of the corresponding genomic sequences, and both prohormone genes were predicted from the same genomic region using Wise2.

A third calcitonin family match in the cattle genome corresponded to a complete cattle calcitonin gene ([SwissProt:B5UBG1]) that contains the previously reported calcitonin peptide ([Swiss-Prot:P01260]), a complete sheep calcitonin gene ([Swiss-Prot:P01261]), and the UniGene cluster Bt.14302. A prohormone gene was recovered by Wise2 using the sheep sequence and the genomic region of the UMD_1.5 assembly. The same protein sequence was also obtained using two human calcitonin variants ([GenBank:NP_001029124] and [GenBank:NP_001732]). A different sequence was obtained from the same region using another human calcitonin variant ([GenBank:NP_001029125]), suggesting that this gene could undergo alternative splicing. This region also had a strong match to the human calcitonin pseudogene, implying that this calcitonin gene was present before the evolutionary split that ultimately originated the human and cattle species.

The last cattle genome match of the calcitonin family corresponded to UniGene cluster Bt.60861, that was associated with a predicted gene ([GenBank:XP_001253111]). The predicted gene contained the domain associated with the Calc_CGRP_IAPP gene superfamily. The goat calcitonin receptor-stimulating peptide-2 ([Swiss-Prot: B3IWF8]) provided the best BLAST match (E-value < 10^-28^) followed by the cattle *CALC3 *(E-value < 10^-27^) to this predicted cattle gene. Although the genomic region is homologous to the other matches, there is no candidate prohormone gene that shows sufficient homology to the predicted gene.

### Prohormone gene expression across tissues and developmental stages

A census of the expression of 62 prohormone genes available in UniGene offered a first glimpse of the cattle transcript profiles. The most frequently expressed prohormone genes (and percentage of reports) were adrenomedullin (*ADM*; 7%), insulin-like growth factor 2 (*IGF2*; 7%), apoptosis-inducing, TAF9-like domain 1 (*APITD1*; 5%), esophageal cancer-related gene 4 protein (*ECRG4*; 5%), secretogranin II (*SCG2*; 5%), platelet-derived growth factor alpha polypeptide (*PDGFA*; 4%), proenkephalin (PENK; 4%), proopiomelanocortin (*POMC*; 4%), chromogranin B (*CHGB*; 3%), endothelin 1 (*EDN1*; 3%), *TOR2A *(3%), and somatostatin (*SST*; 3%). The UniGene expression reports were almost equally distributed across the three developmental stages (fetus, calf and adult) with the fetus and adult having the highest and lowest number of reports, respectively. There were 26 tissues or body sites that exhibited expression of at least one prohormone gene. Most reports of tissues or body sites (and percentage of reports across tissues) were in the brain (12%), kidney (12%) intestine (8%), extraembryonic tissue (7%), ovary (7%) and liver (6%). The distribution across tissues and developmental stages reflects the limited experiments and microarray platforms used. The lack of Unigene expression reports for other prohormone genes are most likely due to incomplete study of cattle neuropeptide genes across tissues and stages.

### Microarray analysis

The analysis of the expression levels of prohormone gene reporters from two cattle microarray experiments indicated that all prohormone genes present in the platform were detected [see Additional file 2]. Two prohormone genes, platelet-derived growth factor beta polypeptide (*PDGFB*) and cortistatin (*CORT*) were significantly (False Discovery Rate adjusted P-value < 0.05) differentially expressed in both studies. In the liver study, cows with ketosis had a 27% higher fold change in *PDGFB *levels and a 50% lower fold change in *CORT *than healthy cows. In the placentome study, IVF embryos had a 45% higher expression of *PDGFB *compared to AI embryos, but there were no significant differences between NT and either AI or IVF embryos. For *CORT*, NT embryos had at least a 50% fold decrease in expression compared to both AI and IVF, but there was no significant difference between AI and IVF. The findings on *PDGFB *confirm reports of high expression levels in the placenta and the important role of this growth factor in stimulating adjacent cells to grow [41]. The expression of *CORT *in a subset of GABAergic cells in the cortex and hippocampus has been associated with synaptic transmission, and furthermore, *CORT *binds to somatostatin receptor subtypes and inhibits cAMP [42].

Six prohormone genes, apelin (*APEL*), chromogranin A (*CMGA*), *EDN1*, insulin (*INS*), neuromedin B (*NMB*), and tachykinin 3 (*TAC3*), were significantly differentially expressed only in the placentome study. Nineteen prohormone genes, *ADM*, natriuretic peptide precursor type C (*ANFC*), *ECRG4*, gastrin (*GAST*), ghrelin/obestatin prepropeptide (*GHRL*), motilin (*MOTI*), arginine vasopressin (*AVP*), neuropeptide FF-amide peptide (*NPFF*), *NPY*, pyroglutamylated RFamide peptide (*QRFP*), *PPY*, proprotein convertase subtilisin/kexin type 1 inhibitor (*PCSK1N*), *PDGFA*, prodynorphin (*PDYN*), prolactin releasing hormone (*PRRP*), *PYY*, *CHGB*, *SCG2*, and *SST *were differentially expressed only in the liver study. Of the remaining 25 prohormone genes, nine had differential expression for other non-embryo type factors in the placentome study. The expression of the *CALCA*, glucagon (*GCG*), and osteocrin (*OSTN*) prohormone genes in the placentome study and natriuretic peptide precursor type A (*NPPA*) in the liver study failed to surpass the expression level of the background, indicating that these prohormone genes were either not expressed or were present in quantities too low to be reliably detected.

### Proprotein convertases

It is critical to assess the presence of proprotein or prohormone convertase enzymes that cleave the prohormone proteins in the cattle genome because a change in these proteases could affect the presence or abundance of a neuropeptide. The mammalian proprotein convertase complement includes furin (*FURIN*), proprotein convertase subtilisin/kexin type 1 (*PCSK1*), proprotein convertase subtilisin/kexin type 2 (*PCSK2*), proprotein convertase subtilisin/kexin type 4 (*PCSK4*), proprotein convertase subtilisin/kexin type 5 (*PCSK5*), proprotein convertase subtilisin/kexin type 6 (*PCSK6*), proprotein convertase subtilisin/kexin type 7 (*PCSK7*), proprotein convertase subtilisin/kexin type 9 (*PCSK9*) and membrane-bound transcription factor peptidase site 1 (*MBTPS1*) [1,12]. Only the cattle *PCSK1 *([SwissProt:Q9GLR1]) and *PCSK2 *([SwissProt:Q9GLR0]) sequences are available. In this study, the complete sequences of *FURIN*, *PCSK1*, *PCSK4*, *PCSK5*, *PCSK7*, *MBTPS1 *and the 7B2 or secretogranin V gene (*SCG5*), which is essential for *PCSK2 *function [43-45], were recovered using the same approach as the prohormone discovery. The complete *PCSK2 *and *PCSK6 *sequences could not be recovered in the Btau_3.1 assembly but were recovered in the Btau_4.0 assembly. Table 2 provides supporting evidence for the presence of the proprotein convertases based on records in the Entez Gene, Unigene and UniProt databases. A partial match of 70 residues to the human *PCSK9 *protein sequence of 690 amino acids was detected but subsequent searches in the cattle EST and trace archives did not support the presence of cattle *PCSK9*. Utilizing the information of the introns and exons from human *PCSK9*, the chromosomal region containing that contained the partial match was found to contain multiple stop codons in different reading frames, suggesting that this gene has been lost from the cattle genome (Dr. Steven Salzberg, Dr. Liliana Florea and Finn Hanrahan, personal communication).

**Table 2:** Inventory of the cattle prohormone convertases across multiple repositories

Entrez Gene Symbol	Entrez Gene Full Name	Entrez Gene ID	Unigene Cluster	Accession Number^1^
*FURIN*	furin (paired basic amino acid cleaving enzyme)	281374	Bt.21338	Q28193
*PCSK1*	proprotein convertase subtilisin/kexin type 1	281967	Bt.5562	Q9GLR1
*PCSK2*	proprotein convertase subtilisin/kexin type 2	281968	Bt.5563	Q9GLR0
*PCSK4*	proprotein convertase subtilisin/kexin type 4	508744	Bt.77029	XP_585571
*PCSK5*	proprotein convertase subtilisin/kexin type 5	528098	Bt.91475	XP_606509
*PCSK6*	proprotein convertase subtilisin/kexin type 6	524684	Bt.62498	XP_603014
*PCSK7*	proprotein convertase subtilisin/kexin type 7	515398	Bt.62043	XP_869877
*SCG5*	secretogranin V (7B2 protein)	508224	Bt.65187	Q2HJG0
*MBTPS1*	membrane-bound transcription factor peptidase, site 1	511682	Bt.18986	Q08E55

^1 ^UniProt or GenBank accession number.

### Prohormone cleavage prediction

The prohormone cleavage prediction models developed by Tegge et al. [17] were applied to 44 newly identified prohormone genes with complete sequences. These sequences excluded the 41 cattle prohormone genes used to develop the cleavage prediction models [17]. Although there were 34% more sites in the new prohormone genes than in the prohormone genes used to develop the predictive models (831 sites compared to 621 sites), the correct classification rate of sequence positions into cleaved and non-cleaved sites was over 86% and the area under the receiver operating characteristic curves was over 76% (Table 3). The amino-acids-plus-properties models provided slightly more true-positive predictions (predicted cleavage sites that were confirmed by empirical data) but slightly more false-positive predictions (predicted cleavages sites that have not yet been reported) than the amino-acids-only models. The artificial neural networks provided slightly higher correct classification rates than the logistic regression models.

**Table 3:** Accuracy of different cleavage prediction models developed on cattle neuropeptides

	Cleavage Prediction Model			
	AA^1^		AA-Prop^2^	
Model Accuracy Statistic	LR^3^	ANT^4^	LR	ANT
True Negative	669	691	667	690
True Positive	44	43	50	46
False Negative	41	42	35	39
False Positive	77	55	79	56
Correct Classification Rate (%)	86	88	86	89
Sensitivity (%)	52	51	59	54
Specificity (%)	90	93	89	92
Area under receiver operating characteristic curve (%)	76	76	82	78

^1^AA = Models developed using only amino acids

^2 ^AA-Prop = Models developed using amino acids plus amino acid physiochemical properties

^3^LR = Models developed using logistic regression

^4 ^ANT = Models developed using artificial neural networks

The cleavage prediction models were useful in evaluating the differences between prohormone sequences predicted from the genome information and those reported in the literature or databases like UniProt. The sequences of 14 prohormone genes detected in the genome differed from previously reported sequences. While these were not used in comparing model performance, these sequence differences resulted in 35 locations with a basic amino acid that had a different probability of prediction of cleavage between the published and predicted sequences. However, the differences in probability of cleavage were typically 0.1 or lower and none of these differences resulted in a different prediction of cleavage.

## Conclusion

Neuropeptides are essential regulators of biological processes including development and growth. The release of the cattle genome sequence has provided a unique opportunity to improve our knowledge of cattle neuropeptides so influential in many biological processes. This first genomic survey of the prohormone gene complement in cattle was conducted using an integrated bioinformatics approach that combines empirical and inferred genomic, transcriptomic and proteomic information to achieve a comprehensive characterization of the cattle neuropeptidome. This approach was able to address and resolve complications that arise from alternative splicing, differential processing of the prohormone, and non-neuropeptide genes that hinder the experimental confirmation and functional characterization of neuropeptides. The cattle neuropeptide census was complemented with a genome-wide characterization of prohormone gene expression profiles and prediction of prohormone cleavage sites that could result in neuropeptides.

Complementary bioinformatic searches of genome and EST resources identified 92 cattle prohormone genes and one prohormone gene not found in the cattle genome. The understanding of the neuropeptide gene complement was substantially advanced because 28 out of the 92 prohormone genes either had no prior experimental evidence at the protein level or only the nucleotide sequence was available, and 9 prohormone genes lacked EST data.

Several findings at the genomic and transcriptomic levels are notable. The bioinformatics approach uncovered a putative *TAC4 *gene that has not been reported in cattle and the isoform of the cattle *TOR2A *gene that contained the putative cattle alpha- and beta-salusin neuropeptides. The integrated strategy also uncovered a potential novel duplication in the calcitonin family and galanin-like pseudogene. There were no matches on the cattle genome for the known mammalian relaxin 1 gene, indicating that this gene has been lost in the cattle genome. The complete sequences for genes *INSL5 *and *RLN3 *recovered in this study have not previously been reported in cattle. With the exception of *PCSK9*, all of the proprotein convertase enzymes that cleave the proprotein sequences were located in the cattle genome, suggesting that *PCSK9 *has been lost from the cattle genome.

The available EST expression information offered an introductory view to the expression patterns of prohormone genes. Of the 62 prohormone genes that have expression reports, *ADM *and *IGF2 *were the most frequently reported. This information was complemented with investigation of the expression profile of prohormone genes in two different microarray studies. The analysis of these studies confirmed the association between prohormone gene expression patterns and reproductive and nutritional processes. There were 8 prohormone genes differentially expressed among placentomes from different embryo types and 21 prohormone genes were differentially expressed in the liver of cows under different nutritional levels.

Available logistic and artificial neural network models had high accuracy (86% correct classification rate) in predicting the cleavage sites of prohormone sequences that result in peptides. The performance was particularly outstanding considering that these models were developed on independent data not used to evaluate the prediction accuracy. Models that include all prohormone data available are being developed. These models will constitute a powerful tool to annotate prohormones on other species related to cattle but without genome sequence information or extensive empirical data to support the development of models. Cattle prohormone gene sequences and neuropeptide prediction approaches are available at . This resource will facilitate the functional characterization of the neuropeptides in cattle and related species with no genome sequence and/or limited neuropeptide studies.

## Authors' contributions

BRS implemented the integrated bioinformatics approach to search for prohormone genes across multiple databases, identified prohormone gene probes in the microarray platform, predicted the cleavage sites on the prohormone sequences that result in putative neuropeptides, interpreted the results, and drafted the manuscript. SRZ analyzed the microarray experiments, helped interpret the results and write the manuscript. JVS obtained funding for the study, participated in its conception, coordination, interpretation of results, and reviewed the manuscript. All authors have read and approved the final version of this manuscript.

## Supplementary Material

Additional file 1 **Cattle prohormone sequences with cleavage data**. Predicted sequences of cattle prohormone genes with cleavage data in NeuroPred format. Click here for file

Additional file 2 **Inventory of the cattle prohormones across multiple repositories and microarray analysis**. Inventory of cattle prohormone genes with accession numbers of major sequence repositories and results of the microarray analysis. Click here for file
