# Supplementary material for: Characterization of the prohormone complement in cattle using genomic libraries and cleavage prediction approaches
Source: BMC Genomics. 2009 May 16;10:228. doi: 10.1186/1471-2164-10-228 (PMC2698874; doi:10.1186/1471-2164-10-228)
Supplement: Additional file 1 — Cattle prohormone sequences with cleavage data. Predicted sequences of cattle prohormone genes with cleavage data in NeuroPred format. [file 1471-2164-10-228-S1.pdf]

Additional File 1: Cattle prohormone sequences with cleavage data.
The sequence and cleavage information is provided for each prohormone in the format used by Neuropred (http://neuroproteomics.scs.uiuc.edu/neuropred.html). This is a modified FASTA format that includes three lines for each prohormone: the sequence title, the precursor amino acid sequence, and a binary indicator of cleavage (1) or non-cleavage (0) for each amino acid in the sequence. The FASTA title includes the length of signal peptide (SignalP=x) that is used by the Neuropred application to treat the first x amino acids of the sequence as part of the signal peptide.

```
>ADM2 Bos taurus adrenomedullin 2 XP_876306 SignalP=24
MARLLTVTLGCISLLYLQLPGALSLSLAGSRPPTGHREPPARTPASGSQPQYPAALPAVWKLHQALQPKKSASLVPARGQPL
RNGPRRHLGPRRPRAQLLRVGCALGTCQVQNLSHRLWQLVGSAGPRDSAPVDPSSPHSYG
0000000000000000000000000000000000000000000000000000000000000000000000000000000000
000000000001000000100000000000000000000000000000000000000000
>ADM Bos taurus adrenomedullin O62827 SignalP=21
MKLVPVALMYLGSLAFLGVDTARLDVAAEFRKKWNKWALSRGKRELRESSSYPTGLADVKAGPVQTLIRPQDVKGASRSPQA
SSPDAARIRVKRYRQSLNNFQGLRSFGCRFGTCTVQKLAHQIYHFTDKDKDGSAPRSKISPQGYGRRRRRSLPEAGLGRTLL
QPPEPKLRGAPDSRVHQVLATLRI
0000000000000000000000000000000000000000000100000000000000000000000000000000000000
0000000000010000000000000000000000000000000000000000000000000000000001000000000000
000000000000000000000000
>NPPA Bos taurus natriuretic peptide precursor A P07501 SignalP=24
MGSSAITVSFLLFLAFQLPGQTGANPVYGSVSNADLMDFKNLLDRLEDKMPLEDEAVPSQVLSEQNEEAGAPLSPLSEMPPW
MGEVNPAQREGGVLGRGPWESSDRSALLKSKLRALLTAPRSLRRSSCFGGRMDRIGAQSGLGCNSFRYRR
0000000000000000000000000000000000000000000000000000000000000000000000000000000000
0000000000000000000000000000000000000001000000000000000000000000000000
>NPPB Bos taurus natriuretic peptide precursor B XP_585559 SignalP=26
MDPQTALSRALLLLLFLHLSLLGCRSHPVGGPGPVSELPGLQELLDRLRDRVSELQAEQLRVEPLQQGQGLEETWDSPAAAP
AGFLGPHHSILRALRGPKMMRDSGCFGRRLDRIGSLSGLGCNVLRRY
0000000000000000000000000000000000000000000000000000000000000000000000000000000000
00000000000000000100100000000000000000000000000
>NPPC Bos taurus natriuretic peptide precursor C P55206 SignalP=23
MHLSQLLACALLLALLSLRPSEAKPGAPPKVPRTPSGEEVAEPQAAGGGQKKGDKTPGGGGANLKDDRSRLLRDLRVDTKSR
AAWTRLLHEHPNARKYKGGNKKGLSKGCFGLKLDRIGSMSGLGC
0000000000000000000000000000000000000000000000000000000000000000000000001000000000
00000000000000100000010000000000000000000000
>APLN Bos taurus apelin Q9TUI9 SignalP=22
MNLRRCVQALLLLWLCLSAVCGGPLLQTSDGKEMEEGTIRYLVQPRGPRSGPGPWQGGRRKFRRQRPRLSHKGPMPF
00000000000000000000000000000000000000000000010010000000000000010000000000000
>CALC Bos taurus Calcitonin (LOC514876) B5UBG1 SignalP=25
MGFGKSSPFLAFSILVLCQAGSLQATPLRSALETLPDPGALSEKEGRLLLAALVKAYVQRKTNELEQEEEQEETEDSSLDGS
RAKRCSNLSTCVLSAYWKDLNNYHRFSGMGFGPETPGKKRDVANSLERDHSFHFGVPQDAN
0000000000000000000000000000000000000000000000000000000000000000000000000000000000
0000000000000000000000000000000000000000000000000000000000000
>CALC2 Bos taurus Calcitonin gene-related peptide 2 precursor  (LOC784945)
XP_001253111 SignalP=25
MGFSKLPLFLVLSMLIIHQAGMLQAAPFRSVWKNGLVPATLTEEESYFLLATMVKYYVQKASELEYETEDFGIIAQERTSNA
ATGMTHKMAGFLGKSGSKIKRNIMSTNVAPKPLAGTTGIFRTK
0000000000000000000000000000000000000000000000000000000000000000000000000000000000
0000000000000000000000000000000000000000000
>CALC3 Bos taurus calcitonin-related polypeptide 3 Q0VBW3 SignalP=25
MGFWKFLPFLVLSFLAVYHAGMLQAAPFRSALENDFDPAILTEKEMCLLLAAMVNNYVQMKTSELKPETEAFRITAQKRSCN
RATCVIHKVAGSLSRSGSEIKRNFMSTNAFGRAAGTFRTKQ
0000000000000000000000000000000000000000000000000000000000000000000000000000001000
00000000000000000000000000000000000000000
>CARTPT Bos taurus CART prepropeptide Q68RJ9 SignalP=27
MESPRLRLLPLLGAALLLLLPLLGALAQEDAELQPRALDIYSAVEDASHEKELIEALQEVLKKLKSKRIPIYEKKYGQVPMC
```

DAGEQCAVRKGARIGKLCDCPRGTSCNSFLLKCL
0000000000000000000000000000000000000000000000000000000000000000000100000000000000
0000000000000000000000000000000000
>CCK Bos taurus cholecystokinin P41520 SignalP=20
MNRGVCLCLLMAVLAAGALAQPMPHADPTGPRAQQAEEAPRRQLRAVPRVDDEPRAQLGALLARYIQQARKAPSGRMSVIKN
LQSLDPSHRISDRDYMGWMDFGRRSAEEFEYTS
0000000000000000000000000000000000000000000010000000000000000001000001000000000000
000000001000100000000001000000000
>C12orf39 Bos taurus chromosome 12 open reading frame 39 Q0VC44 SignalP=26
MKGFKSLVVMTLTLFLVFSFMGNCNSAPQRLFERRNWTPQAMLYLKGAQGRRFLSDQSRRKDLSDRPPLERRSPNSQQLTLP
EAAAVLLAFLQKPQEAGDENLDQTRFLEDSLLNW
0000000000000000000000000000000000100000000000000001000000000000000000000000000000
0000000000000000000000000000000000
>CHGA Bos taurus chromogranin A (parathyroid secretory protein 1) P05059
SignalP=18
MRSAAVLALLLCAGQVIALPVNSPMNKGDTEVMKCIVEVISDTLSKPSPMPVSKECFETLRGDERILSILRHQNLLKELQDL
ALQGAKERTHQQKKHSSYEDELSEVLEKPNDQAEPKEVTEEVSSKDAAEKRDDFKEVEKSDEDSDGDRPQASPGLGPGPKVE
EDNQAPGEEEEAPSNAHPLASLPSPKYPGPQAKEDSEGPSQGPASREKGLSAEQGRQTEREEEEEKWEEAEAREKAVPEEES
PPTAAFKPPPSLGNKETQRAAPGWPEDGAGKMGAEEAKPPEGKGEWAHSRQEEEEMARAPQVLFRGGKSGEPEQEEQLSKEW
EDAKRWSKMDQLAKELTAEKRLEGEEEEEEDPDRSMRLSFRARGYGFRGPGLQLRRGWRPNSREDSVEAGLPLQVRGYPEEK
KEEEGSANRRPEDQELESLSAIEAELEKVAHQLEELRRG
0000000000000000000000000000000000000000000000000000000000000000000000000000000000
0000000000000100000000000000000000000000000000000000000001000000000000000000001000
0000000000000000000000000100000000000000000000010000000000000000000000000000000000
0000000000000000001000000000000000000000000000000000000000000000000100000000000000
0000100000000000000010000000000000000000000000000000000100000000000000000000000000
000000000000000000000000000000000000000
>POMC Bos taurus proopiomelanocortin P01190 SignalP=26
MPRLCSSRSGALLLALLLQASMEVRGWCLESSQCQDLTTESNLLACIRACKPDLSAETPVFPGNGDEQPLTENPRKYVMGHF
RWDRFGRRNGSSSSGVGGAAQKREEEVAVGEGPGPRGDDAETGPREDKRSYSMEHFRWGKPVGKKRRPVKVYPNGAEDESAQ
AFPLEFKRELTGERLEQARGPEAQAESAAARAELEYGLVAEAEAEAAEKKDSGPYKMEHFRWGSPPKDKRYGGFMTSEKSQT
PLVTLFKNAIIKNAHKKGQ
0000000000000000000000000000000000000000000000000000000000000000000000000001000000
0000000100000000000000100000000000000000000000000100000000000000000100000000000000
0000000100000000000000000000000000000000000000000100000000000000000001000000000000
0000000000000000000
>CORT Bos taurus cortistatin XP_001250131 SignalP=19
MLPLCLLLPLLLLPSGATTALSPEGGLAGHESGHMQEVAEIKTNSLLTFLAWWYEWASQARAVPFVGGEAREVSKRQEGAPL
HQSTRQDKTPCKNFFWKTFSSCK
0000000000000000000000000000000000000000000000000000000000000000000000000001000000
00000001000000000000000
>CRH Bos taurus corticotropin releasing hormone Q95MI6 SignalP=24
MRLPLLVSVGVLLVALLPSPPCRALLSRGPIPGARQASQHPQPLXFFQPPPQPQEPQALPTLLRVGEEYFLRLGNLDETRAA
PLSPAASPLASRSSSRLSPDKVAANFFRALLQPRRPFDSPAGPAERGTENALGSRQEAPAARKRRSQEPPISLDLTFHLLRE
VLEMTKADQLAQQAHXNRKLLDIAGK
0000000000000000000000000000000000000000000000000000000000000000000000000000000000
0000000000000000000000000000000000000000000000000000000000000000100000000000000000
00000000000000000000000000
>CRSP1 Bos taurus Calcitonin receptor-stimulating peptide 1 Q75V95 SignalP=25
MGFWKFPPFLVLSILVLYQAGMFHAAPFRSVFDGRFDPATLDEEESRLLLAAMVNDYEQMRARESEKAQKTEGSRIQKRACN
TATCMTHRLAGWLSRSGSMVRSNLLPTKMGFKIFNGPRRNSWF
0000000000000000000000000000000000000000000000000000000000000000000000000000001000
0000000000000000000000000000000000000010000
>C2orf40 Bos taurus chromosome 2 open reading frame 40 Q32KM8 SignalP=31
MANSSARPAFLVMTALALLLLLCVGPGGISGNKLKLLLRKREAPAPTMTPVAVQESRAKEFLSSLRRPKRQLWDRSRPEVQQ
WYQHFLYLGFDEAKFEDDISYWLNRNRNGHDYYDYYQRHYDEDSAIGPRSAHSFRHGASVNYDDY
0000000000000000000000000000000000000000000000000000000000000000000001000000000000
00000000000000000000000000000000000000000000000000000000000000000

>EDN1 Bos taurus endothelin 1 P17322 SignalP=25
MDYFPMIFALLFVAFQGAPEAAVLGTELSAGAEDGGEKPAPATPWRPRRSKRCSCSSLMDKECVYFCHLDIIWVNTPEHVVP
YGLGSPSRSKRSLKDFFPTKATVHRKRCQCASQTDKKCWNFCQAGKELRDQDSMEKAWNNQKRGKDCSKLGEKCLHQQLVAG
RKTRRLEAISNSIKTSFRVAKLKAQLYRDKKVIYNRAH
0000000000000000000000000000000000000000000000000001000000000000000000000000000000
0000000000000000000000000000000000000000000000000000000000000000000000000000000000
00000000000000000000000000000000000000
>EDN2 Bos taurus endothelin 2 Q867A9 SignalP=23
MPTALCSIALALLVALHEGKSQAATTPIPEQPAPLPRARGSHLRTRRCSCSSWLDKECVYFCHLDIIWVNTPGQTAPYGLGN
PPRRRRRSLPRRCECYSARDPACATFCHQRPWTDAVAVPGSGSPAAAFQDGKTQATAGELLQRLRVISATKIHFARQQQKPT
RETRPTHSRQRKR
0000000000000000000000000000000000000000000000100000000000000000000000000000000000
0000000000000000000000000000000000000000000000000000000000000000000000000000000000
0000000000000
>EDN3 Bos taurus endothelin 3 A6QLQ7 SignalP=18
MELGLCFLFGLAVTSAAGWVPHPQSGDAGRSSMPRAPSAATSEGDTKETVATMAARGPSPRSPWREQGPSQFGKQGAEGVPV
HHRARRCTCFTYKDRECVYYCHLDIIWINTPERTAPYGLSSYRGSGSRGRRSAGQFPQSPQPVKGTLRCACTESDDDACLQF
CTWSLAARGNSRSAETSDKEVGKQTGGALGGVRPRR
0000000000000000000000000000000000000000000000000000000000000000000000000000000000
0000010000000000000000000000000000000000000000000000000000000000000000000000000000
000000000000000000000000000000000000
>GAL Bos taurus galanin prepropeptide P11242 SignalP=19
MPRGSVLLLASLLLAAALSATLGLGSPVKEKRGWTLNSAGYLLGPHALDSHRSFQDKHGLAGKRELEPEDEARPGSFDRPLA
ENNVVRTIIEFLTFLHLKDAGALERLPSLPTAESAEDAERS
0000000000000000000000000000000100000000000000000000000000000001000000000000000000
00000000000000000000000000000000000000000
>GALP Bos taurus galanin-like peptide NA SignalP=23
VPLSVRLLLLAVLLSLAETPASAPVHGGRGGWTLNSAGYLLGPVLPSPPRADRGRKEKTTLGILDLWRTIDGLPYSQSLLAS
RSLGETAAKAETGDPGVLGQKVLKEEDAWVPRCSQTSAPNLCPSGSF
0000000000000000000000000000000000000000000000000000000000000000000000000000000000
00000000000000000000000000000000000000000000000
>GAST Bos taurus gastrin P01352 SignalP=21
MQRLCAHVLILVLALAAFCEASWKPHSHLQDAPVAPGANRGQEPLRMNRLGPASNPRRQLGLQDPPHMVADLSKKQGPWVEE
EEAAYGWMDFGRRSAEEGDQHP
0000000000000000000000000000000000000000000000000000000001000000000000000010000000
0000000000001000000000
>GHRL Bos taurus ghrelin/obestatin prepropeptide Q9BDJ6 SignalP=23
MPAPWTICSLLLLSVLCMDLAMAGSSFLSPEHQKLQRKEAKKPSGRLKPRTLEGQFDPEVGSQAEGAEDELEIRFNAPFNIG
IKLAGAQSLQHGQTLGKFLQDILWEEAEETLANE
0000000000000000000000000000000000000000000000000100000000000000000000000100000000
0000000000000000100000000000000000
>GIP Bos taurus gastric inhibitory polypeptide XP_588333 SignalP=21
MLAMKTFSLLVVSLLLAVVLGEKEEDHSKLGFHSKASGSQPRATRYAEGTFISDYSIAMDKIRQQDFVNWLLAQKGKKSDWI
HNITQREAGALELAHQSNRKEEAKEKQGSLPKNPSDEDLLKHLLIRELLAWMVDQMELCRLRFQ
0000000000000000000000000000000000000000000010000000000000000000000000000000000000
0000010000000000000000000000000000000000000000000000000000000000
>GCG Bos taurus glucagon P01272 SignalP=20
MKSLYFVAGLFVMLVQGSWQRSLQNTEEKSSSFPAPQTDPLGDPDQINEDKRHSQGTFTSDYSKYLDSRRAQDFVQWLMNTK
RNKNNIAKRHDEFERHAEGTFTSDVSSYLEGQAAKEFIAWLVKGRGRRDFPEEVNIVEELRRRHADGSFSDEMNTVLDSLAT
RDFINWLLQTKITDRK
0000000000000000000000000000000000000000000000000001000000000000000000000000000000
1000000010000010000000000000000000000000000000010000000000000010000000000000000000
0000000000000000
>GNRH1 Bos taurus gonadotropin-releasing hormone 1 (luteinizing-releasing hormone) Q0VBW7 SignalP=23
MKPTPKLLAGLILLILCVVGCSGQHWSYGLRPGGKRNAENVIDSFQEIAKEVDQPVEPKCCGIVHQSHSPLRDLKAALESL
IEEETGQRKI
0000000000000000000000000000000000010000000000000000000000000000000000000000000000

0000000000
>GNRH2 Bos taurus gonadotropin-releasing hormone 2 XP_001255678 SignalP=30
MGAALLWATFGLGLLLLLLLLTTYPGPSKAQHRSHGWYPGGKRASSLPRDPQHPPRPPAQSPGQIAHTLPSDALAWPEDSVP
WKSRTMTRWLLRGKQHLVQTLLINWTPRATPRSPPIKV
0000000000000000000000000000000000000000001000000000000000000000000000000000000000
00000000000000000000000000000000000000
>GRP Bos taurus gastrin-releasing peptide Q863C3 SignalP=23
MRGREVPLVLLALVLCLAPRGWAAPVTAGRGGALAKMYTRGNHWAVGHLMGKKSVAESPQLHEEESLKEQLREYAQWEEATR
NLLSLLQAKGARGHQMPPWEPLSIHQPAWDSEDVSNFKDTGPQHEGRNPQLN
0000000000000000000000000000000000000001000000000000100000000000000000000000000000
0000000000000000000000000000000000000000000000000000
>HAMP Bos taurus hepcidin antimicrobial peptide Q2NKT0 SignalP=23
MALNTQIRATCLLLLVLLSLTSGSVLPPQTRQLTDLQTKDTAGAAAGLTPVLQRRRRDTHFPICIFCCGCCRKGTCGMCCRT
0000000000000000000000000000000000000000000000000000000010000000000000000000000000
>IAPP Bos taurus islet amyloid polypeptide Q28207 SignalP=22
MGILKLPVVLIVLCVALNHLEGGGKPTESHQMEKRKCGTATCETQRLANFLAPSSNKLGAIFSPTKMGSNTYGKRKKVEILK
REPLSYLPI
0000000000000000000000000000000000010000000000000000000000000000000000000000100000
000000000
>IGF1 Bos taurus insulin-like growth factor 1 (somatomedin C) P07455 SignalP=49
MGKISSLPTQLFKCCFCDFLKQVKMPITSSSHLFYLALCLLAFTSSATAGPETLCGAELVDALQFVCGDRGFYFNKPTGYGS
SSRRAPQTGIVDECCFRSCDLRRLEMYCAPLKPAKSARSVRAQRHTDMPKAQKEVHLKNTSRGSAGNKNYRM
0000000000000000000000000000000000000000000000000000000000000000000000000000000000
000000000000000000000000000000000000010000000000000000000000000000000000
>IGF2 Bos taurus insulin-like growth factor 2 (somatomedin A) P07456 SignalP=24
MGITAGKSVLVLLAFLAFASCCYAAYRPSETLCGGELVDTLQFVCGDRGFYFSRPSSRINRRSRGIVEECCFRSCDLALLET
YCATPAKSERDVSASTTVLPDDVTAYPVGKFFQYDIWKQSTQRLRRGLPAFLRARRGRTLAKELEALREAKSHRPLIALPTQ
DPATHGGASSKASSD
0000000000000000000000000000000000000000000000000000000000000000000000000000000000
0000000001000000000000000000000000000000000000000000000000000000000000000000000000
000000000000000
>INS Bos taurus insulin P01317 SignalP=24
MALWTRLRPLLALLALWPPPPARAFVNQHLCGSHLVEALYLVCGERGFFYTPKARREVEGPQVGALELAGGPGAGGLEGPPQ
KRGIVEQCCASVCSLYQLENYCN
0000000000000000000000000000000000000000000000000000000100000000000000000000000000
01000000000000000000000
>INSL3 Bos taurus insulin-like 3 (Leydig cell) O77801 SignalP=21
MDRRPLTWALVLLGPALAIALGPAAAQEAPEKLCGHHFVRALVRLCGGPRWSSEEDGRPVAGGDRELLRWLEGQHLLHGLMA
SGDPVLVLAPQPLPQASRHHHHRRATAINPARHCCLSGCTRQDLLTLCPH
0000000000000000000000000000000000000000000000000000000000000000000000000000000000
00000000000000000000000100000000000000000000000000
>INSL5 Bos taurus insulin-like 5 XP_869400 SignalP=20
MRGFIFMLFTFSVMLAISEARREGSRKLCGSEYVRTVIYICASSRWRRHAGALLQGQQAERGNHFQLPNEQEISEERAAHNL
PKMDFAGEESLQGEQLPTEGLWRSKKHLVMSRQDLQTLCCTEGCSMSDLSTLC
0000000000000000000000000000000000000000000000010000000000000000000000000000000000
00000000000000000000000000000001000000000000000000000
>INSL6 Bos taurus insulin-like 6 Q32L79 SignalP=19
MPRFLYLCLGLLLVRVSRELNDISRARRLCGQHLLHEIVKLCGDVNWSHVEKEKPFTQLLSQASEKVESFIPDRSESSQTTF
PVWRRATNPVPTSASQEEAINNMKMQSLPEYQYKKANLPFKTRQEESSSSQDISPYIHEIIEFQKKNTNKIKTLSNLFWGNH
PQRKRRGYSEKCCQKGCTKEELIIACLPYIDYENLRKHQQL
0000000000000000000000000000000000000000000000000001000000000000000000000000000000
0000000000000000000000000000000000000000000000000000000000000000000000000000000000
00000100000000000000000000000000000010000
>KISS1 Bos taurus KiSS-1 metastasis-suppressor XP_872566 SignalP=17
MNVLLSWQLMLLLCATAFRETLEKVVPMENPRTTGSQLGPATLRAPWEQSPRCAAGKPEVAGPRPRGAALCPPESSAGPQRL
GPCAPRSRLIPSPRGAVLVQREKDVSAYNWNSFGLRYGRRQAALPGSRSAARG
0000000000000000000000000000000000000000000000000000000000000000010000000000000000
00000000000000000000000000000000000000010000000000000

>PMCH Bos taurus pro-melanin-concentrating hormone A1Z2Z3 SignalP=21
MAKMSFSSYILILTFSLLSQGISLSASKSIRNLDDDMVFKTLRLGKAFQKEDTAEKSIVVPSLEQYKNDESSFMNDEENKNS
KNAGSKHNFLNHGLPLNLAIKPYLALKGSVAFPAENEVQNTESTQEKREIGDEENSAKFPIGRRDFDMLRCMLGRVYRPCWQ
V
0000000000000000000000000000000000000000000000000000000000000000000000000000000000
0000000000000000000000000010000000000000000000001000000000000000100000000000000000
0
>MLN Bos taurus motilin O62820 SignalP=25
MLSRKATAVLLAVHAAAMLASQTEAFVPIFTYGEVRRMQEKERYKGQKKSLSVQQRSEEVGPVDPTEPWEEKQEVIKLTAPV
EIGMRMNSRQLEKYQATLEGLLREVLPPSRNAQ
0000000000000000000000000000000000000000000000001000000000000000000000000000000000
000000000000000000000000000000000
>OXT Bos taurus oxytocin, prepropeptide P01175 SignalP=19
MAGSSLACCLLGLLALTSACYIQNCPLGGKRAVLDLDVRTCLPCGPGGKGRCFGPSICCGDELGCFVGTAEALRCQEENYLP
SPCQSGQKPCGSGGRCAAAGICCSPDGCHEDPACDPEAAFSQH
0000000000000000000000000000001000000000000000000000000000000000000000000000000000
0000000000000000000000000000000000000000000
>AVP Bos taurus arginine vasopressin P01180 SignalP=19
MPDATLPACFLSLLAFTSACYFQNCPRGGKRAMSDLELRQCLPCGPGGKGRCFGPSICCGDELGCFVGTAEALRCQEENYLP
SPCQSGQKPCGSGGRCAAAGICCNDESCVTEPECREGVGFPRRVRANDRSNATLLDGPSGALLLRLVQLAGAPEPAEPAQPG
VY
0000000000000000000000000000001000000000000000000000000000000000000000000000000000
0000000000000000000000000000000000000000000010000000000000000000000000000000000000
00
>NTS Bos taurus neurotensin P01156 2
MMAGMKIQLVCMILLAFSSWSLCSDSEEEMKALETDLLTNMHTSKISKASVPSWKMSLLNVCSLINNLNSQAEETGEFHEEE
LITRRKFPAALDGFSLEAMLTIYQLQKICHSRAFQHWELIQEDILDAGNDKNEKEEVIKRKIPYILKRQLYENKPRRPYILK
RGSYYY
0000000000000000000000000000000000000000000000000000000000000000000000000000000000
0000000000000000000000000000000000000000000000000000000000010000000100000000000000
100000
>NMB Bos taurus neuromedin B Q2T9U8 SignalP=24
MTLRAVGVRLLGGLLLFALLAAGAAPLGWDLPESRSRASKIRVHPRGNLWATGHFMGKKSLEPPSPSLLGTAPHTSLRDQTP
QLSHHLLRVLLQKQALGMSLSVPAPNTQHRRLLVQTLQK
0000000000000000000000000000000000000000000001000000000000100000000000000000000000
000000000000000000000000000000000000000
>NMS Bos taurus neuromedin S Q0VBW8 SignalP=25
MKYLAQFPSILAIYCFCLLQIPSSGFPRPLADASDGLDIVKFEQMAYWASLSRQPKDNQDIYKRFLFHYSRTQEPAHPVKTG
FPPVHPLMRLAAKLADRRMKTFWRRDSRATAADFTKKDYTATLGRPFFLFRPRNGRNLDFDTW
0000000000000000000000000000000000000000000000000000000000000001000000000000000000
000000000000000001000000000000000000000000000000000000010000000
>NMU Bos taurus neuromedin U XP_001250700 SignalP=37
MLRAASRRPEPPAGHVAAGSPLLLLLLLSCCADACGGAPVLPQGLQPEQELQLWKQINDACLSLLSMHPQPQASNALEEICL
TIMRTLPKPQETDEKDNTKRFLFHYSKTQKLGNSNVVSSVLHPLLQLVPQLHERRMKRFRLDEEFQGPIASQSRRYFLFRPR
NGRRSEGYI
0000000000000000000000000000000000000000000000000000000000000000000000000000000000
0000000000000000000000000000000000000000000000000000001001000000000000000000000000
000100000
>NPB Bos taurus neuropeptide B Q8MJV4 SignalP=24
MAGPAMLVAAALALCLLLASPGLAWYKPTAGQGYYSVGRAAGLLSGFHRSPYARRSEPRGGTRSLGGVGTFREMRPNLRSLA
VCVEEVTPNLQSCEPLPDGRATFQCKADVFLSLSASDCRK
0000000000000000000000000000000000000000000000000000001000000000000000000000000000
0000000000000000000000000000000000000000
>NPFF Bos taurus neuropeptide FF-amide peptide precursor Q9TUX7 SignalP=22
MDARQAAALLLVLLLVTDWSHAEGPGGRDGGDQIFMEEDSGAHPAQDAQTPRSLLRSLLQAMQRPGRSPAFLFQPQRFGRNT
RGSWSNKRLSPRAGEGLSSPFWSLAAPQRFGKK
0000000000000000000000000000000000000000000000000000000000000000001000000000000000
000000000001000000000000000000000

>NPS Bos taurus neuropeptide S P0C0P5 SignalP=23
MIGSLKFNFILFLLISTMHMFWCHPISSSKVPGKSDYFVILLNSCPTRMDRRVGLDFLKPILEKTLMKRSFRNGVGTGMKKT
SFRRAKS
0000000000000000000000000000000000000000000000000000000000000000000010000000000000
0000000
>NPW Bos taurus neuropeptide W BT30001-PA SignalP=35
MARGTRVRGPGRGATASRRLLALLWLLLLPPSAGAWYKHVASPRYHTVGRAAGLLMGLRRSPYMWRRAAGPLTWDTLGLGAL
PQGPSARSTLSPGPAALDALLLPSGAQRPWEARRRSSGAGLRVSAPRNLPAPESARQPEWRPGAYSWILAE
0000000000000000000000000000000000000000000000000000000000010000001000000000000000
00000000000000000000000000000000000000000000000000000000000000000000000
>NPY Bos taurus neuropeptide Y Q6RUW3 SignalP=28
MLGSKRLGLSGLTLALSLLVCLGALAEAYPSKPDNPGEDAPAEDLARYYSALRHYINLITRQRYGKRSSPETLISDLLMRES
TGNIPRTRLEDPSMW
0000000000000000000000000000000000000000000000000000000000000000001000000000000000
000000000000000
>HCRT Bos taurus hypocretin (orexin) neuropeptide precursor XP_609741 SignalP=33
MNPSSTKVSWATVTLLLLLLLLPPALLSPGAAAQPLPDCCRQKTCSCRLYELLHGAGNHAAGILTLGKRRPGPPGLQGRLQR
LLQASGNHAAGILTMGRRAGAEPALRPCSGRRCPSEAASSVAPGGRSGV
0000000000000000000000000000000000000000000000000000000000000000000010000000000000
0000000000000000010000000000000000000000000000000
>OSTN Bos taurus osteocrin A5PKG1 SignalP=27
MLDWRLASAHFILAMTLMLWSSGKVFSVGVTTEAFDSGVLGVQSSPTVREAKSATDLAAKLLLLDELVSLENDVIETKKKRS
FSGFGSPLDRLSAGSVSHKGKQRKVVDHPKRRFGIPMDRIGRNRLSNSRG
0000000000000000000000000000000000000000000000000000000000000000000000000000000010
00000000000000000000000000000000000000000000000000
>QRFP Bos taurus pyroglutamylated RFamide peptide P83862 SignalP=18
MRSPYSLPYLLFLPLGACFPVLDTEEPVDAVGGTGREMSWMDPARGRPFPWGSPGWPRAPYPHALLVTAKELRASGKARAGF
QLRLGRQDDGSEATGLLLGEAEKVGGLLGTLAEELNGYSRKKGGFSFRFGRR
0000000000000000000000000000000000000000000000000000000000000000000000000000000000
0000010000000000000000000000000000000000000000000000
>ADCYAP1 Bos taurus adenylate cyclase activating polypeptide 1 (pituitary) A4FV02 SignalP=24
MTMCSGARLALLVYGILMHSSVYGSPAASGLRFPGIRPENEVYDEDGNPQQDFYDSESLGVGSPASALRDAYALYYPAEERD
VAHGILNKAYRKVLDQPSARRSPADAHGQGLWDPGGSADDDSEPLSKRHSDGIFTDSYSRYRKQMAVKKYLAAVLGKRYKQ
RVKNKGRRIPYL
0000000000000000000000000000000000000000000000000000000000000000000000000000000010
0000000000000000000000000000000000000000000000001000000000000000000000000000001000
000000010000
>PPY Bos taurus pancreatic polypeptide P01302 SignalP=29
MAAAHRCLFLLLLSTCVALLLQPPLGALGAPLEPEYPGDNATPEQMAQYAAELRRYINMLTRPRYGKRDKEGTLDFLECGSP
HSAVPRYGKRDKEGTLDFLECGSPHSAVPRWVFSLSCVPRCLGQENGGV
0000000000000000000000000000000000000000000000000000000000000000000100000000000000
0000000001000000000000000000000000000000000000000
>PCSK1N Bos taurus proprotein convertase subtilisin/kexin type 1 inhibitor A4IFR2 SignalP=33
MAGSPLLHGPRAGGVGLLVLLLLGLLGPPRTLCARPVKEPRSLGAASPPLAEASGHRRFRRAAPRGEAAGAVQELARALAHL
LEAERQERARAEAQEAEDQQARVLAQLRRIWGAPRTSDPALGLENDPDAPAAQLARALLRARLDPAALAAQLVPAPAPAAAL
RLRPPVYDDGPTGPDGEDAGDETPDLDPELLRYLLGRILTGIADTEAVAAPRRLRRAADQDTGPELPPEGVLGALLRVKRLE
TPAPQAPARRLLP
0000000000000000000000000000000000000000100000000000000000001000000000000000000000
0000000000000000000000000000000000000000000000000000000000000000000000000000000000
0000000000000000000000000000000000000000000000000000000100000000000000000000100100
0000000001000
>PDGFA Bos taurus platelet-derived growth factor alpha polypeptide Q2KJ15 SignalP=20
MRTWACLLLLGCGYLANALAEEAEIPREVIERLAHSQIHSIRDLQRLLEIDSVGAEEPLETSLRAHGGHGAKHALEKRPVPI
RRKRSIEEAIPAVCKTRTVIYEIPRSQVDPTSANFLIWPPCVEVKRCTGCCNTSSVKCQPSRVHHRNVKVAKVEYFRKKAKL
KEVQVRLEEHLECTCTSASPSPDHREEEAGRRRESGKKRKRKRLKPT

0000000000000000000000000000000000000000000000000000000000000000000000000000000000
0001000000000000000000000000000000000000000000000000000000000000000000000000000000
00000000000000000000000000000000000000000000000
>PDGFB Bos taurus platelet-derived growth factor beta polypeptide (simian sarcoma viral (v-sis) oncogene homolog) B1H0W5 SignalP=20
MNRCWALFLSLCCYLRLVSAEGDPIPEELYKMLSDHSIRSFDDLQRLLHGDSVDEDGAELDLNLTRSHSGGELESLSRGRRS
LGSPTVAAEPAVIAECKTRTEVFEISRRLIDRTNANFLVWPPCVEVQRCSGCCNNRNVQCRPTQVQDRKVQVKKIEIVRKKK
IFKKATVTLVDHLACRCETVVARAVTRTPGSSQEQRARTPQTRVTIRTVRVRRPPKGKHRKFKHTHDKTALKETLGA
0000000000000000000000000000000000000000000000000000000000000000000000000000000010
0000000000000000000000000000000000000000000000000000000000000000000000000000000000
00000000000000000000000000100000000000000000000000000000000000000000000000000
>PDGFD Bos taurus platelet derived growth factor D A4IFC0 SignalP=23
MHRLVLVYTLVCANFCSYRDTSATPQSASIKALRNANLRRDDLYRRDETIEVTGHGHVQSPRFPNSYPRNLLLTWRLHSQEK
TRIQLAFDNQFGLEEAENDICRYDFVEVEDISETSTVIRGRWCGHKEVPPRIISRTNQIKITFKSDDYFVAKPGFKIYYSFV
EYFQPAAASETNWESVTSSISGISYHSPSVTDPTLTADALDKTIAEFDTVEDLLKHFNPESWQEDLENLYLDTPHHRGRSYH
DRKSKVDLDRLNDDVKRYSCTPRNYSVNLREELKLTNVVFFPRCLLVQRCGGNCGCGTVNWKSCACNSGKTVKKYHEVLKFE
PGYFKRRGRAKHMALVDIQLDHHERCDCICSSRPPR
0000000000000000000000000000000000000000000000000000000000000000000000000000000000
0000000000000000000000000000000000000000000000000000000000000000000000000000000000
0000000000000000000000000000000000000000000000000000000000000000000000000000100000
0000000000000000000000000000000000000000000000000000000000000000000000000000000000
000000000000000000000000000000000000
>PDYN Bos taurus prodynorphin Q95104 SignalP=20
MVWQGLVLAACLLALPSVTADCLAQCSLCAVKTQDGPQPINPLVCSLECQAALQPAQEWERCQGLLSFLTPFTVGLNGKEDL
KSKTVLEESSSEPAKHIRPYLKELEKNRFLLSTPAEKNALSSSLVEKLRGLSGRLGEDAESELMGDAQLNDGALEAEARDSN
EEEPKEQVKRYGGFLRKYPKRSSEVTGKEAGEGEGGEVGHEDLYKRYGGFLRRIRPKLKWDNQKRYGGFLRRQFKVVTRSQE
DPSAYYEELFDV
0000000000000000000000000000000000000000000000000000000000000000000000000000000000
0000000000000000000000000000000000000000000000000000000000000000000000000000000000
0000000001000000000010000000000000000000000001000000000000000000100000010000001000
000000000000
>PENK Bos taurus proenkephalin P01211 SignalP=24
MARFLGLCTWLLALGPGLLATVRAECSQDCATCSYRLARPTDLNPLACTLECEGKLPSLKTWETCKELLQLTKLELPPDATS
ALSKQEESHLLAKKYGGFMKRYGGFMKKMDELYPLEVEEEANGGEVLGKRYGGFMKKDAEEDDGLGNSSNLLKELLGAGDQR
EGSLHQEGSDAEDVSKRYGGFMRGLKRSPHLEDETKELQKRYGGFMRRVGRPEWWMDYQKRYGGFLKRFAEPLPSEEEGESY
SKEVPEMEKRYGGFMRF
0000000000000000000000000000000000000000000000000000000000000000000000000000000000
0000000000000100000010000001000000000000000000000100000010000000000000000000000000
0000000000000000100000000010000000000000100000010000000000001000000100000000000000
00000000010000000
>PNOC Bos taurus prepronociceptin O62647 SignalP=19
MKILFCDLLLLSLFSSVSSSCQKDCLVCREKLRPTLDSFSLEMCILECEEKAFTSPLWTPCTKVMARGSWQLSPADPDHVAA
ALDQPRASEMQHLKRMPRVRSLFQRQKRTEPGLEEVGEIEQKQLQKRFGGFTGARKSARKLANQKRFSEFMRQYLVLSMQSS
QRRRTLHQNGNA
0000000000000000000000000000000000000000000000000000000000000000000000000000000000
0000000000000010000000000000000000000000000000100000000000000000010000000000000000
000100000000
>PROK2 Bos taurus prokineticin 2 Q863H5 SignalP=27
MRSSRCARLLLLLLLPPLLLTPPAGDAAVITGACDRDPQCGGGMCCAVSLWVKSIRICTPMGKVGDSCHPMTRKNHFGNGRQ
ERRKRKRRRKKKVPFLGRRMHHTCPCLPGLACSRTSFNRYTCLAQK
0000000000000000000000000000000000000000000000000000000000000000000000000000000000
0000000000000000000000000000000000000000000000
>PRLH Bos taurus prolactin releasing hormone P81264 SignalP=22
MKAVGAWLLCLLLLGLALQGAASRAHQHSMEIRTPDINPAWYAGRGIRPVGRFGRRAAPGDGPRPGPRRVPACFRLEGGAE
PSRALPGRLTAQLVQE
0000000000000000000000000000000010000000000000000000000100000000000000000000000000
0000000000000000
>PTHLH Bos taurus parathyroid hormone-like hormone P58073 SignalP=24

MLWRLVQQWSVAVFLLSYSVPSCGRSVEELGRRLKRAVSEHQLLHDKGKSIQDLRRRFFLHHLIAEIHTAEIRATSEVSPNS
KPAPNTKNHPVRFGSDDEGKYLTQETNKVETYKEQPLKTPGKKKKSKPGKRKEQEKKKRRTRSAWLTSYVAGTGLEEDYLSD
ISATSLELNSRRH
0000000000000000000000000000000000010000000000000000000000000000000000000000000000
0000000000000000000000000000000000000000000000000000000000010000000000000000000000
0000000000100
>PTH Bos taurus parathyroid hormone P01268 SignalP=25
MMSAKDMVKVMIVMLAICFLARSDGKSVKKRAVSEIQFMHNLGKHLSSMERVEWLRKKLQDVHNFVALGASIAYRDGSSQRP
RKKEDNVLVESHQKSLGEADKADVDVLIKAKPQ
0000000000000000000000000000001000000000000000000000000000000000000000000000000000
000000000000000000000000000000000
>PYY Bos taurus peptide YY P51694 SignalP=28
MMSGRRSWPAMATVLLTLLVCLGELVDAYPAKPQAPGEHASPDELNRYYTSLRHYLNLVTRQRFGKRDFSEALLSILLFPDR
EDPPVKSRPEGAYLW
0000000000000000000000000000000000000000000000000000000000000000001000000000000000
000000000000000
>PYY2 Bos taurus peptide YY, 2 (seminalplasmin) P06833 SignalP=32
MMAGRRSWPAMATVLLALLVCLGELVDSKPQPSDEKASPDKHHRFSLSRYAKLANRLANPKLLETFLSKWIGDRGNRSVK
00000000000000000000000000000000000000000000000000000000000000000000000000000000
>RLN3 Bos taurus relaxin 3 BT10002-PA SignalP=25
MVRHLVLLLLALGVLTGELLPRTEARATAYGVKLCGREFIRAVIFTCGGSRWRRAHDAVEDTFPEVDTDSESELDEAVASSE
WLALTKSPQAFYGGRQDWQGTPGSLRGRRDVLAGLSSSCCKRGCSKSEIGRLC
0000000000000000000000000000000000000000000000000000010000000000000000000000000000
00000000000000000000000000001000000000000000000000000
>RESP18 Bos taurus regulated endocrine-specific protein 18 A0JNL8 SignalP=29
MQRQLWLGGFRGLWLLVCFLLLNSRLGGSSDVSGHDGQSQVGVGQLWPLPGFTTPVFKHLQVLLQQIMPHDLFWKDDMTQEV
MTQKMGRTSKLHPEDPCVRSGPAAFPTRTPGVRGKQEEKLRLLFPKSPMVKVNKDQCFTSKVVSKVLKHEVANPVKGFFESP
PTVGHNLVAD
0000000000000000000000000000000000000000000000000000000000000000000000000000000000
0001000000000000001000000000000000000000000000000000000000000000000000000000000000
0000000000
>NPVF Bos taurus neuropeptide VF precursor Q9GM96 SignalP=21
MEIISLKRFILLMLATSSLLTSNIFCTDESRMPNLYSKKNYDKYSEPRGDLGWEKERSLTFEEVKDWAPKIKMNKPVVNKMP
PSAANLPLRFGRNMEEERSTRAMAHLPLRLGKNREDSLSRWVPNLPQRFGRTTTAKSITKTLSNLLQQSMHSPSTNGLLYSM
ACQPQEIQNPGQKNLRRRGFQKIDDAELKQEK
0000000000000000000000000000000000000000000000000000000010000000000000000000000000
0000000000010000010000000000000100000001000000000010000000000000000000000000000000
00000000000000000000000000000000
>CHGB Bos taurus chromogranin B (secretogranin 1) P23389 SignalP=20
MQPAALLGLLGATVVAAVSSMPVDIRNHNEEVVTHCIIEVLSNALLKSSAPPITPECRQVLKKNGKELKNEEKSENENTRFE
VRLLRDPADTSEAPGLSSREDSGEGDAQVPTVADTESGGHSRERAGEPPGSQVAKEAKTRYSKSEGQNREEEMVKYQKRERG
EVGSEERLSEGPGKAQTAFLNQRNQTPAKKEELVSRYDTQSARGLEKSHSRERSSQESGEETKSQENWPQELQRHPEGQEAP
GESEEDASPEVDKRHSRPRHHHGRSRPDRSSQEGNPPLEEESHVGTGNSDEEKARHPAHFRALEEGAEYGEEVRRHSAAQAP
GDLQGARFGGRGRGEHQALRRPSEESLEQENKRHGLSPDLNMAQGYSEESEEERGPAPGPSYRARGGEAAAYSTLGQTDEKR
FLGETHHRVQESQRDKARRRLPGELRNYLDYGEEKGEEAARGKWQPQGDPRADENREEARLRGKQYAPHHITEKRLGELLN
PFYDPSQWKSSRFERKDPMDDSFLEGEEENGLTLNEKNFFPEYNYDWWEKKPFEEDVNWGYEKRNPVPKLDLKRQYDRVAEL
DQLLHYRKKSAEFPDFYDSEEQMSPQHTAENEEEKAGQGVLTEEEEKELENLAAMDLELQKIAEKFSGTRRG
0000000000000000000000000000000000000000000000000000000000000000000000000000000000
0000000000000000000000000000000000000000000000000000000000000000000000000000000000
0000000000000000000000000000000000000000000000000000000000000000000000000000000000
0000000000000000000000000000000000000000000000000000000000000000000000000000000000
0000000000000000000000000000000000000000000000000000000000000000000000000000000000
0000000000000000000000000000000000000000000000000000000000000000000000000001000000
0000000000000000000000000000000000000000000000000000000000000000000000000100000000
000000001000000000000000000000000000000000000000000000000000000000000000
>SCG2 Bos taurus secretogranin II (chromogranin C) P20616 SignalP=27
MAEAKTHWLGAVLSLIPLIFLLSEAEAASFQRNQLLQKEPDLRLENVQRFPSPEMIRALEYIEKLRQQAHKEESSPDYNPYQ
GVSVPLQQKENGDLPESSRDSLSEDEWMKIIAEALRQAENEPQSAPKENKPYTLNSEKNFPMDMPDDYETQQWAERKLKHMR

FPPMYEENSRDNPFKRTNEIVEEQYTPQNLATLESVFQELGKLTGPNSQKRERADEEQKLYTDDEDDIYKANNIAYEDVVGG
EDWNPVEEKIESQTQEEVRDSKENADKTEQINDEMKRSGQLGLQDEDLRKESKDQLSDDVSKVITYLKRLVNAAGSGRSQNG
QTGERAIRLFEKPLDPQSIYQLIEISRNLQIPPEDLIDMLKTGEKPVEPEQELEIPVEPEDISEVDLDHPDLFQNKMLSKNG
YPKAPGHAVAEALPEGLSVEDILNLLGMESAANPKPPYFPNQYNREKVLSRLPYGPGRSKANQLPKAVWMPDVENRQMAYEN
LNDKDQELGEYLARMLVKYPEIMNANPAKRVPSQGSTEDDRQDENQIEQALKEHLSQHSSQETDKLASVSKRLPVGTPKSDD
TPNRPYLDEDLLVKVLEYLNQEKAEKGREHIAKRAMENM
0000000000000000000000000000000000000000000000000000000000000000000000000000000000
0000000000000000000000000000000000000000000000000000000000000000000000000000000000
0000000000000001000000000000000000000000000000000010000000000000000000000000000000
0000000000000000000000000000000000000000000000000000000000000000000000000000000000
0000000000000000000000000000000000000000000000000000000000000000000000000000000000
0000000000000000000000000000000000000000000000000000000000000000000000000000000000
0000000000000000000000000000000000000000000000000000000000000000000000000000000000
000000000000000000000000000000000000000
>GHRH Bos taurus growth hormone releasing hormone P63292 SignalP=19
MLLWVFFLVTLTLSSGSHGSLPSQPLRIPRYADAIFTNSYRKVLGQLSARKLLQDIMNRQQGERNQEQGAKVRLGRQVDGVW
TDQQQMALESTLVSLLQERRNSQG
0000000000000000000000000000010000000000000000000000000000000000000000000001000000
000000000000000000000000
>SST Bos taurus somatostatin P26917 SignalP=24
MLSCRLQCALAALSIVLALGGVTGAPSDPRLRQFLQKSLAAAAGKQELAKYFLAELLSEPNQTEIDALEPEDLSQAAEQDEM
RLELQRSANSNPAMAPRERKAGCKNFFWKTFTSC
0000000000000000000000000000000000000000000000000000000000000000000000000000000000
0000010000000000000100000000000000
>PTH2 Bos taurus parathyroid hormone 2 P0C171 SignalP=30
METRQVSRSPRVRLLLLLLLLLVVPWGVRTASGVALPPVGVLSLRPPGRAWADPATPRPRRSLALADDAAFRERARLLAALE
RRHWLNSYMHKLLVLDAP
0000000000000000000000000000000000000000000000000000000000001000000000000000000000
000000000000000000
>TAC1 Bos taurus tachykinin, precursor 1 P01289 SignalP=19
MKILVAVAVIFFISTQLSAEEIGANDDFNYWSDWSDSDQIKEEMPEPFEHLLQRIARRPKPQQFFGLMGKRDADSSIEKQVA
LLKALYGHGQLSHKRHKTDSFVGLMGKRALNSVAYERSVMQDYERRRK
0000000000000000000000000000000000000000000000000000000010000000000000100000000000
000000000000001000000000000100000000000000000000
>TAC4 Bos taurus tachykinin 4 (hemokinin) BT10001-PA SignalP=19
MLLCVTLLLLLGLSACTVAGDKELAINAEVGSWVAVTLEVDVIPSILLQLRNVKKGKASQFFGLMGKQVGGE
000000000000000000000000000000000000000000000000000000000000000000000000
>TAC3 Bos taurus tachykinin 3 P08858 SignalP=20
MRSTLLFAVILALSSARSLGAVCEESQEQVVPGGGHSKKDSNLYQLPPSLLRRLYDSRVVSLDGLLKMLSKASVGPKESPLP
QKRDMHDFFVGLMGKRNLQPDTPVDINQENIPSFGTFKYPPSVE
0000000000000000000000000000000000000000000000000000000000000000000000000000000000
00100000000000010000000000000000000000000000
>TOR2A Bos taurus torsin family 2, member A P0C7W1 SignalP=26
MAAATRSCRPWGSLLGLIWLVSAAAASWDLSSLRCNFGSFCECDFQPDFQGLECDLAQHLAGQHLARSLVVKALKAFLQDPA
PTKPLVLSLHGWTGTGKSYVSSLLAHYLFRDGLRSPHVHHFSPVIHFPHPSHLERYKKDLKSWVQGNLTVCSRSLFLFDEMD
KLAPGLIEVLRPFLGSSWVVYGTNYRKAIFIFIRWLLALGHHGRASPGRSGALPATPAAPRAALCAQRAGPSGPGAQG
0000000000000000000000000000000000000000000000000000000000000000000000000000000000
0000000000000000000000000000000000000000000000000000000000000000000000000000000000
000000000000000000000000001000000000000000000000100000000000000000000000000000
>TRH Bos taurus thyrotropin-releasing hormone A6QM11 SignalP=24
MPGSWFLLAMALTLTLTGVPGGRAQLDVAQQEADLSEQQGLEDLLRQAERLLLLREDLQRLREDQDDSVSESQIFQPDWFSK
RQHPGKREEEEEADEAVEGEGEGGAAGLHKRQHPGRRGDVAAWAVDAGQQKRQHPGRRASWLGYAVTKRQHPGRRLVDPRAQR
SWEEAGEDEDEGGDLMSEKRQHPGKRALGSPCGPRGPCGQASLLLGLLDDLSRGQGAEEKRQHPGRRAAWASEPLEE
0000000000000000000000000000000000000000000000000000000000000000000000000000000000
1000001000000000000000000000100000100000000000000100000100000000001000001000000000
00000000000000000001000001000000000000000000000000000000000010000010000000000
>UCN Bos taurus urocortin Q4AE15 SignalP=24
MKTAGRAALLAALLLLAQLRPGSSQWSPEEEAAAGVRDPRLRWSPGTRNHGGGARALLLLLAERFPRRRAEQGRWGSGTAG

ERPRRDDPPLSIDLTFHLLRTLLELARTQSQRERAEQNRIIFDSVGK
00000000000000000000000000000000000000000000000000000000000000000000000000000000
00001000000000000000000000000000000000000000000
>UCN2 Bos taurus urocortin 2 Q0KK90 SignalP=19
MTRWALLTLMVLTLGRTLLVPATPTPGFQLLPQNFPQATACPVTSESPSGSTTAPSAAWGRPSPDPHPGPRITLSLDVPLGL
LQILLEQARARAVREQAAANARILAQVGRR
00000000000000000000000000000000000000000000000000000000000000000000100000000000
000000000000000000000000000000
>UCN3 Bos taurus urocortin 3 (stresscopin) Q1RMJ9 SignalP=23
MLVPAPFLLVLLLLLGAPQVGLSQRSPKAGSSPSCLHTALREAEKSQRKDTSLLIKRTFPALPRGDPEDQEGQEEEDTEKRT
FPGSVGGGGGGGAGSTRYKYPSQAQFQGRPSQDKANSDRRTKVTLSLDVPTNIMNILFNIAKAKNLRAKAAANAHLMAQIGR
KK
00000000000000000000000000000000000000000000000000000000000000000000000000000000
00000000000000000000000000000000000000001000000000000000000000000000000000000000
00
>UTS2 Bos taurus urotensin 2 XP_582447 SignalP=20
MYKLVSCCLLFIGSLNPLLSLPVLDSRQESLQLLAPEDVRSTLDELERASLLQMLPEMSGAETGEGLRNTDPITNIFYPRGN
MRKAFSGQDPKLFLSDLLSRIRKQSKKRGPSSECFWKYCV
00000000000000000000000000000000000000000000000000000000000000000000000000000000
0000000000000000000000000001000000000000
>UTS2D Bos taurus urotensin 2 domain containing XP_871317 SignalP=28
MNVILSTTLSFGLLTLLSVMIFLESVHGVPYFVQGNELLPDKEDADGEELLLALLNKNFGFQRPSNIDIELADKLEELNQLE
KLKEQLMEAKDAEMSYAIDGLSSSHPNKRDCCCNLTASKITKMKERDRG
00000000000000000000000000000000000000000000000000000000000000000000000000000000
0000000000000000000000000001000000000000000000000
>VEGFC Bos taurus vascular endothelial growth factor C Q9XS50 SignalP=27
MHLLGFCSVACSLLAAALLPGPRRAPAAAAAFESGLGFSDTEPDAGENKAYAGKEMEEQLRSVSSVDELMTVLYPEYWKMY
KCQLRKGGWQHSTEQANTNIRTGETLKFAAAHYNTEILRSIDNEWRKTQCMPREVCIDVGKEFGAATNTFFKPPCVSVYRCG
GCCNSEGQQCMNTSTSYLSKTLFEITVPLSQGPKPVTISFANHTSCRCMSKLDVYRQVHSIIRRSLPAALPQCQAANKTCPA
DYIWNNHVCRCLAQHDFIFSPSAGDDSADGFHDICGPNKELDEETCQCVCKGGLQASSCGPHKELDRDSCQCVCKNKLFPSS
CGANREFDENTCQCICKKTCPRNQPLNPGKCACECTENPQKCFLKGKKFQHQTCSCYRRPCTNRVKHCEQGLSFSEEVCRCV
PSYWKRPHVN
00000000000000000000000000000000000000000000000000000000000000000000000000000000
00000000000000000000000000000000000000000000000000000000000000000000000000000000
00000000000000000000000000000000000000000000000000000000000001000000000000000000
00000000000000000000000000000000000000000000000000000000000000000000000000000000
00000000000000000000000000000000000000000000000000000000000000000000000000000000
0000000000
>FIGF Bos taurus c-fos induced growth factor (vascular endothelial growth factor D) A7MBB6 SignalP=21
MYRQWAVVNIFMMSSLQLVQGSSYERGPVKRASRSMLERSEQQIRAASGLEELLQITHFEDWKLWRCRLKLKSLTSTDSRSA
SHRSTRFAATFYDMETLKVIDEEWQRTQCSPRETCVEVASELGSSTDTFFKPPCVNVFRCGGCCNEESLVCVNTSTSYVSKQ
LFEISVPLTSVPELVPVKVANHTGCKCFPTAPRHPFSIIRRSIQIPEEDRCSHSKKLCPVDMLWDSNKCKCVLQEENPLAGM
EDHTHLQELALCGQHMKFDEDRCECVCKTPCPRDLIQHPENCSCIECRESLESCCQKHKIFHPDTCSCEDRCPFRPRTCANG
KPACPKHCRFPKEKRATHGLQDRENP
00000000000000000000000000000000000000000000000000000000000000000000000000000000
00000100000000000000000000000000000000000000000000000000000000000000000000000000
00000000000000000000000000000000000000010000000000000000000000000000000000000000
00000000000000000000000000000000000000000000000000000000000000000000000000000000
00000000000000000000000000
>VIP Bos taurus vasoactive intestinal peptide P81401 SignalP=25
METRSKPQLLVFLTLFSVLFSQTLAWPLFGAPSALRMGDRIPFEGANEPDQVSLKADTDILQDALAENDTPYYDVSRNVRHA
DGVFTSDYSRLLGQLSAKKYLESLIGKRVSNSISEDQGPIKRHSDAVFTDNYTRLRKQMAVKKYLNSILNGKRSSEGESPDF
LEELEK
00000000000000000000000000000000000000000000000000000000000000000000000000000000
00000000000000000000000000100000000000001000000000000000000000000000000000001000000000
000000
