# Supplementary material for: Characterization of the prohormone complement in cattle using genomic libraries and cleavage prediction approaches
Source: BMC Genomics. 2009 May 16;10:228. doi: 10.1186/1471-2164-10-228 (PMC2698874; doi:10.1186/1471-2164-10-228)
Supplement: Additional file 2 — Inventory of the cattle prohormones across multiple repositories and microarray analysis. Inventory of cattle prohormone genes with accession numbers of major sequence repositories and results of the microarray analysis. [file 1471-2164-10-228-S2.pdf]

Additional File 2. Inventory of the cattle prohormones across multiple sequence repositories and microarray analysis.

Entrez Gene			Accession Number[1]	Unigene Cluster[2]	Microarray Analysis		
Symbol	Full name	Gene ID			GPL2853 ProbeID[3]	Liver[4]	Plac.[5]
ADCYAP1	adenylate cyclase activating polypeptide 1 (pituitary)	615187	A4FV02	Bt.56289	-[6]	-	-
ADM	adrenomedullin	280713	O62827	Bt.2047	OLIGO_10789	0.1	-
ADM2	adrenomedullin	618896	XP_876306	-	-	-	-
APLN	apelin	282143	Q9TUI9	Bt.36587	OLIGO_01877	-	0.05
AVP	arginine vasopressin	280728	P01180	Bt.49210	OLIGO_12228	0	-
C12orf39	chromosome 12 open reading frame 39 (MGC143392)	509493	Q0VC44	Bt.17644	-	-	-
CALC	calcitonin (LOC514876)	514876	B5UBG1	Bt.14302	-	-	-
CALC2	calcitonin gene-related peptide 2 precursor (LOC784945)	784945	XP_001253111	Bt.60861	-	-	-
CALC3	calcitonin-related polypeptide 3	614663	Q0VBW3	Bt.73268	-	-	-
CRSP1	calcitonin receptor-stimulating peptide 1	407218	Q75V95	Bt.29881	OLIGO_11795	-	-
CARTPT	cocaine and amphetamine responsive transcript	281071	Q68RJ9	Bt.20015	OLIGO_00906	-	-
CCK	cholecystokinin	280755	P41520	Bt.30532	OLIGO_10925	-	-
CHGA	chromogranin A (parathyroid secretory protein 1)	515133	P05059	Bt.49630	OLIGO_12255	-	0
CHGB	chromogranin B (secretogranin 1)	281137	P23389	Bt.5448	OLIGO_12256	0.1	-
CORT	cortistatin	781658	XP_001250131	Bt.20197	OLIGO_06471	0.05	0.05
CRH	corticotropin releasing hormone	319094	Q95MI6	Bt.37342	-	-	-
C2orf40	esophageal cancer related gene 4 protein	280799	Q32KM8	Bt.26513	OLIGO_07373	0.1	-
EDN1	endothelin 1	280802	P17322	Bt.59529	OLIGO_12259	-	0.01
EDN2	endothelin 2	281191	Q867A9	Bt.12942	OLIGO_12203		-
EDN3	endothelin 3	513753	A6QLQ7	Bt.26462	-	-	-
FIGF	c-fos induced growth factor (vascular endothelial growth factor D)	286799	A7MBB6	Bt.13096	-	-	-

GAL	galanin prepropeptide	280799	P11242	Bt.176	OLIGO_11825	-	-
GALP	galanin-like peptide putative psuedogene	-	-	-	-	-	-
GAST	gastrin	280800	P01352	Bt.312	OLIGO_11826	0.05	-
GCG	glucagon	280802	P01272	Bt.410	OLIGO_11827	-	-
GHRH	growth hormone releasing hormone	281191	P63292	Bt.45045	OLIGO_12245	-	-
GHRL	ghrelin/obestatin prepropeptide	281192	Q9BDJ6	Bt.8142	OLIGO_11891	0.1	-
GIP	gastric inhibitory polypeptide	511073	XP_588333	Bt.62398	-	-	-
GNRH1	gonadotropin-releasing hormone 1	768325	Q0VBW7	Bt.74220	-	-	-
GNRH2	gonadotropin-releasing hormone 2	788698	XP_001255678	-	-	-	-
GRP	gastrin-releasing peptide	615323	Q863C3	Bt.59634	OLIGO_04190	-	-
HAMP	hepcidin antimicrobial peptide	512301	Q2NKT0	Bt.19804	-	-	-
HCRT	hypocretin (orexin) neuropeptide precursor	281222	XP_609741	Bt.13040	-	-	-
IAPP	islet amyloid polypeptide	100138011	Q28207	AJ675853	-	-	-
IGF1	insulin-like growth factor 1 (somatomedin C)	281239	P07455	Bt.12750	OLIGO_13252	-	-
IGF2	insulin-like growth factor 2 (somatomedin A)	281240	P07456	Bt.98687	OLIGO_11385	-	-
INS	insulin	280829	P01317	Bt.453	OLIGO_11833	-	0.05
INSL3	insulin-like 3 (Leydig cell)	281870	O77801	Bt.91145	OLIGO_09151	-	-
INSL5	insulin-like 5	613480	XP_869400	Bt.101509	-	-	-
INSL6	insulin-like 6	774023	Q32L79	Bt.95012	-	-	-
KISS1	KiSS-1 metastasis-suppressor	615613	XP_872566	Bt.88121	-	-	-
MLN	motilin	280860	O62820	Bt.35	OLIGO_11840	0.05	-
NMB	neuromedin B	506584	Q2T9U8	Bt.10630	OLIGO_12713	-	0.1
NMS	neuromedin S	768331	Q0VBW8	Bt.74246	-	-	-
NMU	neuromedin U	782109	XP_001250700	-	-	-	-
NPB	neuropeptide B	280880	Q8MJV4	Bt.12289	OLIGO_11842	-	-
NPFF	neuropeptide FF-amide peptide precursor	281354	Q9TUX7	Bt.220	OLIGO_11915	0.1	-
NPPA	natriuretic peptide precursor A	281355	P07501	Bt.29967	OLIGO_01450	-	-
NPPB	natriuretic peptide precursor B	508734	XP_585559	Bt.30348	OLIGO_01485	-	-

NPPC	natriuretic peptide precursor C	281356	P55206	Bt.4102	OLIGO_03837	0.01	-
NPS	neuropeptide S	613540	P0C0P5	Bt.38215	-	-	-
NPVF	neuropeptide VF precursor	281451	Q9GM96	Bt.7042	OLIGO_11931	-	-
NPW	neuropeptide W	NPW	BT30001-PA	DY084317	-	-	-
NPY	neuropeptide Y	504216	Q6RUW3	Bt.33726	OLIGO_00570	0.05	-
NTS	neurotensin	280881	P01156	Bt.440	OLIGO_11843	-	-
OSTN	osteocrin	511114	A5PKG1	Bt.63450	OLIGO_04569	-	-
OXT	oxytocin, prepropeptide	280888	P01175	Bt.183	OLIGO_12229	-	-
PCSK1N	proprotein convertase subtilisin/kexin type 1 inhibitor	513603	A4IFR2	Bt.11657	OLIGO_12573	0.01	-
PDGFA	platelet-derived growth factor alpha polypeptide	505908	Q2KJ15	Bt.18780	OLIGO_08837	0.05	-
PDGFB	platelet-derived growth factor beta polypeptide	540106	B1H0W5	Bt.27755	OLIGO_06645	0.01	0.1
PDGFD	platelet derived growth factor D	525931	A4IFC0	Bt.40070	OLIGO_02271	-	-
PDYN	prodynorphin	281385	Q95104	Bt.124	OLIGO_11921	0.05	-
PENK	proenkephalin	281387	P01211	Bt.166	-	-	-
PMCH	pro-melanin-concentrating hormone	508013	A1Z2Z3	Bt.1190	OLIGO_12940	-	-
PNOC	prepronociceptin	281414	O62647	Bt.199	OLIGO_11924	-	-
POMC	proopiomelanocortin	281416	P01190	Bt.8797	OLIGO_03638	-	-
PPY	pancreatic polypeptide	280900	P01302	Bt.98647	OLIGO_11844	0.05	-
PRLH	prolactin releasing hormone	286856	P81264	Bt.210	OLIGO_12189	0.1	-
PROK2	prokineticin 2	387602	Q863H5	Bt.64118	OLIGO_12286	-	-
PTH	parathyroid hormone	280903	P01268	Bt.95	OLIGO_11845	-	-
PTH2	parathyroid hormone 2	617680	P0C171	-	-	-	-
PTHLH	parathyroid hormone-like hormone	286767	P58073	Bt.12848	OLIGO_01565	-	-
PYY	peptide YY	615800	P51694	Bt.53545	OLIGO_11846	0.01	-
PYY2	peptide YY, 2 (semi-plasmin; LOC280905)	280905	P06833	Bt.324	-	-	-
QRFP	pyroglutamylated RFamide peptide	379045	P83862	Bt.23584	OLIGO_12284	0.01	-
RESP18	regulated endocrine-specific protein 18	510410	A0JNL8	Bt.16279	-	-	-
RLN3	relaxin 3	RLN3	BT10002-PA	BI682322	-	-	-

SCG2	secretogranin II (chromogranin C)	281477	P20616	Bt.5427	OLIGO_11936	0.05	-
SCT	secretin	-	P63296 (Fragment)	Bt.97670	OLIGO_03002	-	-
SST	somatostatin	280932	P26917	Bt.49664	OLIGO_07220	0.01	-
TAC1	tachykinin, precursor 1	281512	P01289	Bt.12930	-	-	-
TAC3	tachykinin 3	281513	P08858	Bt.37192	OLIGO_12260	-	0.05
TAC4	tachykinin 4	-	BT10001-PA	-	-	-	-
TOR2A	torsin family 2, member A	534311	P0C7W1	Bt.22232	OLIGO_09208	-	-
TRH	thyrotropin-releasing hormone	613414	A6QM11	Bt.82069	-	-	-
UCN	urocortin	518336	Q4AE15	Bt.87884	-	-	-
UCN2	urocortin 2	751828	Q0KK90	Bt.76072	-	-	-
UCN3	urocortin 3 (stresscopin)	751573	Q1RMJ9	Bt.60941	-	-	-
UTS2	urotensin 2	506055	XP_582447	Bt.35964	-	-	-
UTS2D	urotensin 2 domain containing	614653	XP_871317	Bt.87093	-	-	-
VEGFC	vascular endothelial growth factor C	282122	Q9XS50	Bt.3913	OLIGO_12077	-	-
VIP	vasoactive intestinal peptide	280956	P81401	Bt.11212	OLIGO_11851	-	-

[1] UniProt, GenBank or Bovine Genome Sequencing and Analysis Consortium (http://bovinegenome.org) accession numbers.

[2] UniGene cluster or GenBank accession number

[3] Microarray Oligonumber refers to the SPOT_ID of the NCBI GEO platform GPL2853.

[4] Unadjusted significance level of treatment effect in the liver gene expression study.

[5] Unadjusted significance level of treatment effect in the placentome gene expression study.

[6] - denotes that there is no information is available.
